# Identification of SpoVID as a putative new substrate of the sporulation-specific protease YabG in *Bacillus subtilis*

**DOI:** 10.3389/fmicb.2026.1838356

**Published:** 2026-08-26

**Authors:** Ritsuko Kuwana, Yasuhiro Kasahara, Kiyoshi Ito, Hiromu Takamatsu

**Affiliations:** 1 Faculty of Pharmaceutical Sciences, Setsunan University, Hirakata, Japan; 2 Institute of Low Temperature Science, Hokkaido University, Sapporo, Japan

**Keywords:** *Bacillus subtilis*, coat, morphogenetic protein, spore, SpoVID, YabG

## Abstract

*Bacillus subtilis* YabG, a cysteine protease expressed in mother cells during sporulation, is involved in the processing of spore coat proteins. However, the full spectrum of YabG substrates and the physiological significance of its proteolytic activity during sporulation remain poorly understood. A combination of SDS–PAGE and LC–MS/MS analysis revealed 83 proteins in *yabG*-deficient spores. Among these, 29 proteins were absent from wild-type spores and considered YabG-specific, including known substrates (such as SafA and SpoIVA) and newly identified candidate substrates, such as SpoVID. To elucidate the role of the YabG protease in spore coat formation in *B. subtilis*, we investigated the assembly of SpoVID onto the spore coat using immunoblotting with an anti-SpoVID antibody and fluorescence microscopy with a SpoVID–GFP fusion protein. Immunoblotting showed that SpoVID exists as a full-length 70-kDa protein in wild-type cells 5 h after sporulation, but was absent from or undetected in wild-type mature spores; however, it was detected as multiple bands in *yabG*-deficient spores. Treatment with a protease inhibitor blocked SpoVID processing in wild-type cells, indicating that YabG and other proteases are required for proteolytic processing during spore maturation. Fluorescence microscopy of GFP fusion proteins demonstrated that YabG was involved in precise SpoVID–GFP localization to the spore coat during spore maturation. Observation of YabG–GFP with 2-(4-aminophenyl) benzothiazole, a membrane stain, from sporulation to the mature spore stage showed that YabG was expressed under the control of SigK and then localized to the inner spore coat after the assembly of the SigE-dependent coat protein was completed. Additionally, in *spoVID*-deficient spores, the YabG–GFP signal at the forespore surface was markedly reduced, likely reflecting a general consequence of impaired spore coat encasement. Finally, our findings suggest that the YabG substrates include the basement layer and inner coat proteins and YabG processes these substrates after localizing to the basement layer and inner coat. We propose that YabG regulates spore coat formation both temporally and spatially by controlling coat protein localization and remodeling the coat structure.

## 1 Introduction

*Bacillus subtilis* is a gram-positive endospore-forming bacterium that undergoes a complex sporulation process in response to nutrient limitation. Mature spores are highly resistant dormant cells surrounded by a multilayered structure known as the spore coat. This coat is composed of more than 70 different proteins that are precisely assembled in a temporal and spatial manner and plays a critical role in protecting spores from environmental stress (Henriques and Moran, 2007; Driks, 1999; Driks and Eichenberger, 2016; Setlow, 2012; McKenney et al., 2013). Coat proteins are expressed in mother cells under the control of sporulation-specific sigma factors, SigE and SigK and are organized into several structural layers, including the basement layer, inner coat, outer coat, and crust. Although early coat assembly is initiated primarily by SigE-dependent proteins, coat formation and maturation continue through sporulation by the coordinated action of both SigE- and SigK-dependent proteins (Driks and Eichenberger, 2016; McKenney et al., 2013).

The proper localization and assembly of coat proteins depends on a hierarchy of morphogenetic proteins. SpoIVA, together with SpoVM, is involved in initial coat protein recruitment to the forespore surface and anchors the coat to the forespore membrane (McKenney et al., 2013; Secaira-Morocho et al., 2020). SpoVID interacts with SpoIVA and is required for spore coat encasement, ensuring that coat proteins uniformly cover the entire forespore surface (Beall et al., 1993; Wang et al., 2009; Nunes et al., 2018). SafA promotes inner coat layer assembly and is recruited to the forespore surface through an interaction with SpoIVA, whereas its proper encasement around the forespore depends on SpoVID (Ozin et al., 2000, 2001; Müllerová et al., 2009). CotE directs outer coat layer assembly and possibly defines the interface between the inner and outer coat, with a possible contribution to crust assembly (Driks, 1999; Driks and Eichenberger, 2016; McKenney et al., 2010, 2013). Together, these morphogenetic proteins establish the spatial framework required for proper coat assembly and maturation.

YabG is a sporulation-specific protease expressed under the control of SigK in mother cells and is localized to the spore coat (Takamatsu et al., 2000b). Previous studies have shown that YabG is involved in the processing of specific spore coat proteins such as SpoIVA, SafA (YrbA), CotF, CotT, GerQ (YwdL), YeeK, and YxeE (Takamatsu et al., 2000b; Kuwana et al., 2006, 2007; Takamatsu et al., 2009). YabG is also required for the correct localization of spore coat proteins, such as YeeK and YxeE, during spore development (Kuwana et al., 2007; Takamatsu et al., 2009). Importantly, SpoIVA and SafA have also been reported to be substrates of YabG, highlighting their central role in regulating early coat morphogenesis (Takamatsu et al., 2000a); however, among the major morphogenetic proteins involved in coat assembly, whether SpoVID and CotE are substrates of YabG has not been examined. The activity of YabG appears to be tightly regulated during sporulation. Immunoblotting analyses detected YabG specifically during mid-to-late sporulation stages (T4–T6), whereas the protein was no longer detectable in mature spores (T18) (Takamatsu et al., 2000a). Several YabG substrates, including SafA, GerQ, and YeeK, have also been implicated in Tgl-dependent cross-linking reactions, suggesting that YabG-mediated proteolysis may function as part of a broader coat maturation pathway (Kuwana et al., 2006; Fernandes et al., 2019). These observations suggest that YabG-mediated proteolysis is temporally controlled and coordinated with spore coat assembly and maturation (Kuwana et al., 2006).

Our recent biochemical study revealed that purified *B. subtilis* YabG functions as an endo-type cysteine protease that hydrolyzes peptide bonds at the carboxyl side of arginine residues in substrate proteins and that YabG has autolytic activity (Yamazawa et al., 2022). YabG is a highly conserved protease in spore-forming bacteria, with homologs present across diverse species (Galperin et al., 2022). Notably, Arg-directed cleavage appears to be a conserved feature of YabG proteases. In *B. subtilis*, YabG undergoes autocleavage at multiple Arg residues (Yamazawa et al., 2022), whereas in *Clostridioides difficile*, YabG cleaves the germination proteins CspBA into CspB and CspA and processes preproSleC into proSleC at sites immediately downstream of Arg residues (Kevorkian et al., 2016; Osborne et al., 2024). In addition to its role in germination protein processing, YabG has been implicated in spore coat and exosporium formation in *C. difficile*. *yabG* disruption affects CotE processing, SpoIVA accumulation, and the expression of the SigK-dependent genes *cotA* and *cdeM*, which are involved in spore surface and exosporium assembly (Marini et al., 2023).

Thus, the function of YabG in spore formation and morphogenesis may represent a conserved mechanism among spore-forming bacteria. However, the full spectrum of YabG substrates and the physiological significance of its proteolytic activity during sporulation remain poorly understood. In particular, why YabG cleaves specific substrate proteins within the developing spore remains unclear. To address these questions, we systematically identified YabG-specific putative substrate proteins and examined their roles in spore morphogenesis. We employed an integrated approach combining sodium dodecyl sulfate-polyacrylamide gel electrophoresis (SDS–PAGE), liquid chromatography-tandem mass spectrometry (LC–MS/MS)-based proteomics, immunoblotting, and fluorescence microscopy using *B. subtilis* wild-type and *yabG*-deficient spores. A comprehensive proteomic analysis of wild-type spore proteins was first reported by Kuwana et al. (2002), who revealed numerous spore-associated proteins. Building on this approach, we analyzed *yabG*-deficient spores to identify proteins that accumulated or whose expressions were altered in the absence of YabG. Although preliminary findings from this analysis have been reported previously (Kuwana, 2009), a detailed characterization of the identified proteins, including their molecular weights, regulation, and potential as YabG substrates, has not been presented.

In the present study, we comprehensively characterized YabG-specific substrate proteins and examined their localization and processing using GFP-tagged constructs. Our results identify SpoVID as a new putative YabG substrate and provide novel insights into the role of YabG-dependent proteolysis in orchestrating spatial and temporal remodeling of the spore coat, particularly through its action on morphogenetic proteins.

## 2 Methods

### 2.1 Bacterial strains, plasmids, media, and general techniques

The *B. subtilis* and *Escherichia coli* strains and plasmids used in this study are listed in Table 1. The *B. subtilis* strains used were derivatives of strain 168, and those constructed in this study were prepared via transformation with plasmid DNA and confirmed using PCR. *E. coli* strain JM109 was used for plasmid production. The oligonucleotides used for PCR amplification are shown in Table 2.

**Table 1:** Bacterial strains and plasmids used in this study.

Strains	Genotype/ description	Source, reference, or construction
***B. subtilis***		
168	*trpC2*	1A1 (*Bacillus* Genetic Stock Center)
S6D5E	*trpC2 spoVID*::pMutin3	Kuwana et al. (2006)
YABG5E	*trpC2 yabG*::pMutin3	Kuwana et al. (2006)
YRBA5E	*trpC2 safA (yrbA)*::pMutin3	Kuwana et al. (2006)
COTB8G	*trpC2 cotB-gfp cat*	Imamura et al. (2010)
COTF8G	*trpC2 cotF-gfp cat*	Imamura et al. (2010)
COTT8G	*trpC2 cotT-gfp cat*	Takamatsu et al. (2009)
S6D8G	*trpC2 spoVID-gfp cat*	This work
YABG8G	*trpC2 yabG-gfp cat*	This work
COTB8GYABG5E	*trpC2 yabG*::pMutin3 *cotB-gfp cat*	This work
COTF8GYABG5E	*trpC2 yabG*::pMutin3 *cotF-gfp cat*	This work
COTT8GYABG5E	*trpC2 yabG*::pMutin3 *cotT-gfp cat*	This work
S6D8GYABG5E	*trpC2 yabG*::pMutin3 *spoVID-gfp cat*	This work
***E. coli***		
JM109	*relA supE44 endA1 hsdR17 gyrA96 mcrA mcrB*+ *thiΔ(lac-proAB)/F'(traD36 proAB*+ *lacIq lacZΔM15)*	Sambrook et al. (1989)
Plasmids		
pGFP7C	*gfp cat*	Kuwana et al. (2006)
pCOTB8G	*cotB-gfp cat*	Imamura et al. (2010)
pCOTF8G	*cotF-gfp cat*	Imamura et al. (2010)
pCOTT8G	*cotT-gfp cat*	Takamatsu et al. (2009)
pS6D8G	*spoVID-gfp cat*	This work
pYABG8G	*yabG-gfp cat*	This work
pMALEH6	*bla lacI tac promoter His6*	Kuwana et al. (2004)
pMCOTD1A	*bla lacI tac promoter malE cotD His6*	This work
pMCOTX2A	*bla lacI tac promoter malE cotX His6*	This work
pMS6D1A	*bla lacI tac promoter malE spoVID His6*	This work
pTUE1122	*bla lacI tac promoter His6*	Nakane et al. (1995)
pTASA1	*bla lacI tac promoter tasA His6*	This work

**Table 2:** Primers used in this study.

Primers	Sequence	Restriction site
S6D602	5′-cggggatccgtgaagaactggagac-3′	BamHI
S6D1724R	5′-acgctcgaggcatggctatttttatattgag-3′	XhoI
YABG236	5′-aatggatccgccaagattataagctg-3′	BamHI
YABG869R	5′-aacctcgagttggacttataaggcatacc-3′	XhoI
COTD3	5′-tttggatccgcatcactgcagaccgc-3′	BamHI
COTD226R	5′-ccactcgagtagtcgcagcaaggttt-3′	XhoI
COTX3	5′-aatggatccggaaagcagaccatattctt-3′	BamHI
COTX327R	5′-attctcgagtctgcaattgatgtcagag-3′	XhoI
S6DM16	5′-attggatccaaaggaggatatgaacttgc-3′	BamHI
S6D1724R	5′-acgctcgaggcatggctatttttatattgag-3′	XhoI
TASAM486	5′-gagctcgagttcaatacaggttcctt-3′	XhoI
TASA783R	5′-ctgagatctatttttatcctcgctatgcg-3′	BglII

In this study, we used a Campbell-type single-crossover recombination method to construct a series of insertion mutants of *B. subtilis* (Errington and Vogt, 1990; Vagner et al., 1998). For GFP fusion strains, DNA fragments encoding the C-terminal region of *spoVID* and *yabG* were used, such that plasmid integration placed *gfp* in-frame at the 3′-end of each gene under the control of its native promoter, preserving the open reading frame of the fusion protein. To construct plasmids carrying a *gfp* fusion, DNA fragments encoding *spoVID* and *yabG* were amplified via PCR. The oligonucleotide primers S6D602 and S6D1724R and YABG236 and YABG869R were used to amplify *spoVID* and *yabG*, respectively. The PCR products were then digested with the restriction enzymes *Bam*HI and *Xho*I, and inserted into the *Bam*HI/*Xho*I-cut plasmid pGFP7C to generate the recombinant plasmids pS6D8G and pYABG8G, respectively (Table 1) (Kuwana et al., 2006). These plasmids were introduced into *B. subtilis* 168 via transformation and selected for chloramphenicol resistance (5 μg/mL), yielding strains S6D8G and YABG8G, respectively (Table 1). Plasmids pCOTB8G, pCOTF8G, pCOTT8G, and pS6D8G were introduced into YABG5E via transformation, and selected for chloramphenicol resistance (5 μg/ml) yielding strains COTB8GYABG5E, COTF8GYABG5E, COTT8GYABG5E, and S6D8GYABG5E, respectively (Table 1) (Imamura et al., 2010; Takamatsu et al., 2009). In each of these strains, *gfp* was inserted in-frame at the 3′-end of the respective gene (*cotB, cotF, cotT*, or *spoVID*) at the native chromosomal locus by Campbell-type single-crossover recombination, such that each protein is expressed as a C-terminal GFP fusion from its native promoter. DNA recombination was confirmed via PCR.

To construct plasmids for the overproduction of the CotD, CotX, and SpoVID proteins as MalE fusion, DNA fragments encoding *cotD, cotX*, and *spoVID* were PCR-amplified with oligo-nucleotide primers COTD3 and COTD226R, COTX3 and COTX327R, S6DM16 and S6D1724R, respectively (Table 2). The amplified fragments were digested with the restriction enzymes *Bam*HI and *Xho*I, and inserted into the *Bam*HI/*Xho*I-digested plasmid pMALEH6 (which contains a 6 × His tag) to generate the recombinant plasmids pMCOTD1A, pMCOTX2A, and pMS6D1A, respectively (Table 1) (Kuwana et al., 2004). To construct plasmids for the overproduction of TasA proteins, DNA fragments encoding *tasA* were PCR-amplified with oligo-nucleotide primers TASAM486 and TASA783R (Table 2). The amplified fragments were digested with the restriction enzymes *Xho*I and Bgl*II*, and inserted into the *Xho*I/*Bgl*II-digested plasmid pTUE1122 (which contains a 6 × His tag) to generate the recombinant plasmids pTASA1 (Table 1) (Nakane et al., 1995). These plasmids were introduced into *E. coli* JM109 and the resulting transformants were verified via PCR.

*B. subtilis* strains were grown in Difco sporulation medium (DS medium) (Schaeffer et al., 1965). *B. subtilis* sporulation was performed as described previously (Kuwana et al., 2002). We used a cOmplete™ Protease Inhibitor Cocktail (PI) (Merck, Tokyo, Japan), to confirm the proteolytical processing of SpoVID *in vivo*, as previously described (Takamatsu et al., 2000a). Recombinant DNA was prepared according to standard protocols (Sambrook et al., 1989). The preparation of competent cells, transformation, and preparation of chromosomal *B. subtilis* DNA were performed as previously described (Cutting and Vander Horn, 1990).

### 2.2 Purification of recombinant MalE–CotD, MalE–CotX, MalE–SpoVID, and TasA proteins from *E. coli*

*E. coli* JM109 carrying the recombinant plasmids pMCOTD1A, pMCOTX2A, and pMS6D1A were grown in 200 ml of Luria broth supplemented with ampicillin (50 μg/ml) at 37 °C for 3 h. The culture was then supplemented with 1 mM isopropyl-β-D-thiogalactopyranoside (FUJIFILM, Tokyo, Japan) and incubated for an additional 3 h at 37 °C. His-tagged recombinant proteins were purified under denaturing conditions. Briefly, the harvested cells were resuspended in 3 mL SDS-sonication buffer (1% SDS, 100 mM sodium phosphate [pH 7.8], 300 mM NaCl) and heated at 100 °C for 5 min. After adding 7 mL same buffer and centrifugation at 10,000 × *g* for 10 min, the supernatant was incubated with Ni-NTA agarose beads (QIAGEN, Tokyo, Japan) at room temperature for 3 h with mixing. The beads were collected by centrifugation, washed three times with SDS-sonication buffer, and bound proteins were eluted twice with 200 μL elution buffer (SDS-sonication buffer supplemented with 100 mM EDTA). The eluates were further purified via electroelution following SDS–PAGE, as previously described (Takamatsu et al., 1998). To remove SDS, the purified proteins were dialyzed against phosphate-buffered saline (PBS; 137 mM NaCl, 2.7 mM KCl, 10 mM Na_2_HPO4, 1.8 mM KH_2_PO4, pH 7.4) and stored at −20 °C until use.

### 2.3 Preparation of antibodies against MalE–CotD, MalE–CotX, MalE–SpoVID, and TasA

Purified MalE–CotD, MalE–CotX, MalE–SpoVID, and TasA (1 mL, 0.2 mg/mL) and 16 mg killed BD Difco *Mycobacterium tuberculosis* cells (Becton, Dickinson and Company, Tokyo, Japan) were mixed with 2 mL BD Difco complete Freund's adjuvant (Becton, Dickinson and Company), and 3 mL each emulsion was injected into healthy rabbits. After 2 weeks, the rabbits were injected with solutions containing MalE–CotD, MalE–CotX, and MalE–SpoVID in BD Difco incomplete Freund's adjuvant (Becton, Dickinson and Company). Two weeks after the second immunization, antibodies against MalE–CotD, MalE–CotX, and MalE–SpoVID were isolated as described previously (Takamatsu et al., 1998). Rabbit antibodies against MalE–CotD, MalE–CotX, MalE–SpoVID, and TasA were collected as described previously (Takamatsu et al., 1998). The specificity of each antibody was verified by immunoblot analysis using the corresponding gene-disruption mutant strain. The immunoreactive band detected in the wild-type strain was not detected in the corresponding mutant strain.

### 2.4 Preparation of spores

*B. subtilis* strains were grown in DS medium at 37 °C, as described previously (Kuwana et al., 2002). Mature spores were harvested 18 h after cessation of exponential growth (T18) and washed once with 10 mM Tris-HCl (pH 7.2). Spore samples were then prepared using the procedure described by Kuwana et al. (2002), with some modifications. To remove cell debris and vegetative cells, the pellets were suspended in 0.1 mL lysozyme buffer (10 mM Tris-HCl [pH 7.2] with 1% [w/v] lysozyme, and 1 × cOmplete™ Protease Inhibitor Cocktail [Merck]) and incubated at 25 °C for 10 min. In some experiments, cOmplete™ Protease Inhibitor Cocktail (Merck, Tokyo, Japan) was added to the lysozyme buffer. The pellets were then washed repeatedly with a buffer (10 mM Tris-HCl [pH 7.2] and 0.5 M NaCl) at room temperature. After this treatment, more than 99% spores were refractive, and almost no dark or gray spores were visible under phase-contrast microscopy. The samples did not contain vegetative cells or cellular debris (data not shown). Finally, the pellets were washed with a buffer (10 mM Tris-HCl, pH 7.2) at room temperature and suspended in 10 mM Tris-HCl (pH 7.2) until subsequent analyses.

### 2.5 Preparation of protein extracts from sporulating cells

Bacterial cultures (10 mL) were harvested during sporulation and washed with 10 mM Tris-HCl (pH 7.2). The pellets were suspended in 100 μL 10 mM Tris-HCl with 1 × cOmplete™ Protease Inhibitor Cocktail (Merck). The pellets were suspended in 100 μL lysozyme buffer (0.4 mg/mL lysozyme and 1 × cOmplete™ Protease Inhibitor Cocktail [Merck] in 10 mM Tris-HCl, pH 7.2), and incubated at 37 °C for 15 min. The protein samples were solubilized in 0.3 mL loading buffer (62.5 mM Tris-HCl [pH 6.8], 10% [w/v] SDS, 10% [v/v] 2-mercaptoethanol, 10% [v/v] glycerol, and 0.05% [w/v] bromophenol blue) and boiled for 5 min (Kuwana et al., 2002).

### 2.6 Solubilization of proteins from mature spores

Spore proteins were extracted by suspending purified spores in 0.1 mL SDS–PAGE loading buffer followed by boiling for 5 min, as described previously (Kuwana et al., 2002). The concentration of extracted proteins was determined using the RC DC Protein Assay Kit (Bio-Rad Laboratories, Tokyo, Japan) according to the manufacturer's instructions. For Coomassie Brilliant Blue R-250 (CBB) staining, 10 μg total spore proteins per lane were separated by SDS–PAGE and stained with CBB as described previously (Takamatsu et al., 2000b). For immunoblot analysis, 2.5 μg total spore proteins per lane were separated by SDS–PAGE and transferred to polyvinylidene difluoride (PVDF) membranes. Equal amounts of protein were loaded in each lane for all analyses.

### 2.7 Immunoblotting

For immunoblotting, proteins were transferred onto a polyvinylidene difluoride (PVDF) membrane (Immobilon; 0.45-μm pore size) (Merck), and then subjected to standard protocols. The resulting blot was incubated with rabbit immunoglobulin G (IgG) against MalE–CgeA, CotA, MalE–CotB, MalE–CotD, CotE, MalE–CotH, CotS, CotSA, MalE–CotX, MalE–CotY, MalE–SpoVID, TasA, and GFP as the primary antibody, and with donkey anti-rabbit immunoglobulin G-horseradish peroxidase conjugate (Cytiva, Tokyo, Japan) as the secondary antibody, as described previously (Kuwana et al., 2005; Takamatsu et al., 2000a; Kakeshita et al., 2001; Kuwana et al., 2004; Takamatsu et al., 1998, 1999; Kuwana et al., 2007). To confirm GFP fusion protein processing, immunoblotting was also performed with an anti-GFP antibody using spore extracts prepared from CotF–GFP, CotT–GFP, and SpoVID–GFP strains in both wild-type and *yabG*-deficient backgrounds (Kuwana et al., 2007). Primary antibodies were used at a dilution of 1:2,500, and donkey anti-rabbit IgG-horseradish peroxidase conjugate (Cytiva, Tokyo, Japan) was used as the secondary antibody at a dilution of 1:10,000. Protein bands were visualized using an Amersham™ ECL™ kit, according to the manufacturer's instructions (Cytiva, Tokyo, Japan). The anti-*E. coli* MalE antibody did not react with any of the spore proteins of *B. subtilis*, as described previously (Kuwana et al., 2004). All immunoblotting experiments were performed at least three times using independent spore or cell preparations, and representative results are shown.

### 2.8 In-gel enzymatic digestion and LC–MS/MS analysis

YabG spore samples were separated via SDS–PAGE, stained with CBB, and YabG-specific bands between 55 kDa and 14.5 kDa were serially divided into 24 slices at approximately 1-mm intervals. Proteins were extracted from the gel slices and identified using LC–MS/MS, as described previously (Kuwana et al., 2002). Protein mass analysis was performed using nanoliquid chromatography combined with nanoelectrospray ion-trapping tandem mass spectrometry (nanoLC–nanoESI–MS/MS) with an LC-Q Deca mass spectrometer (Thermoquest) coupled with a microcapillary nano-flow LC Magic 2002 (Michrom Bioresources). Protein identification was performed using the search program (Thermoquest) (Yates et al., 1995) against the *Bacillus subtilis* Protein Database as described previously (Kuwana et al., 2002).

### 2.9 Effect of protease inhibitors on proteolysis of SpoVID

*B. subtilis* strain 168 cells were cultured in DS medium. A cOmplete™ Protease Inhibitor Cocktail (PI; Merck, Tokyo, Japan) was added to the culture at 5 h after the initiation of sporulation (T5) to a final concentration of 1 × according to the manufacturer's instructions. Wild-type cells were cultured in the presence or absence of PI from T5, and proteins were extracted from cells collected at T18 of sporulation. Samples with (+) or without (–) PI were analyzed by SDS–PAGE. SpoVID and CotE proteins were detected by immunoblotting using anti-SpoVID and anti-CotE antibodies, respectively.

### 2.10 Phase-contrast and fluorescence microscopy

After 24 h of growth on DS medium agar medium at 37 °C, cultures of strains harboring GFP fusion on the chromosome were stained using 2-(4-aminophenyl) benzothiazole (APBT) (final concentration, 0.01 mg/mL) (Tokyo Chemical Industry, Tokyo, Japan) in 50 mM Tris-HCl (pH 7.6) for 10 min at 25 °C. Afterward, the samples were transferred to microscope slides, as previously described (Kuwana et al., 2022). Phase-contrast and fluorescence images of *B. subtilis* cells were obtained using an Olympus BX51 phase-contrast microscope with additional fluorescence tools and mirror cube units (Olympus, Tokyo, Japan). The green fluorescence of GFP was detected using the mirror cube unit U-MGFPHQ, while the blue fluorescence of APBT was detected using the mirror cube unit U-MNUA2. A UPlanApo 100 × oil Iris 3 pH objective lens and a U-TV1X-2 camera adapter were used for detection (Olympus). Images were captured using an ORCA-SPARK digital CMOS camera C11440-36U (Hamamatsu Photonics Inc. Shizuoka, Japan) and analyzed using the CellSens imaging software (Olympus). The exposure time for fluorescence imaging ranged from 0.25 to 4.0 s.

### 2.11 Measurement of spore cell length, fluorescence localization, and fluorescence intensity

Mature spores, characterized by a high refractive brightness, exhibit a dark outer edge under phase-contrast microscopy. To determine the precise location of fluorescence within the spores, we identified the negative peak of the phase-contrast intensity at the boundary between the spore's interior and exterior, following the protocol described by Imamura et al. (2010). Spore length was determined by measuring the distance between the negative peaks observed at both poles of the spore under a phase-contrast microscope. Measurements were performed using CellSens imaging software (Olympus). Fluorescence microscopy of the spore coat and cortical proteins fused with GFP revealed a ring-shaped fluorescence pattern along the periphery of the spores. Based on the method described by Imamura et al. (2010), fluorescence localization was quantified by identifying the positive fluorescence peaks of the GFP fusion protein at both poles of the spore's long axis. The distance between the two positive peaks was calculated using CellSens software. Thirty spores were analyzed for each sample and the following distances were measured: negative peak distance (spore length from phase-contrast microscopy images) and positive peak distance (fluorescence intensity peaks from fluorescence microscopy images using fluorescent reagents and/or GFP fusion proteins). Relative fluorescence localization in the spores was computed as the average of these measured distances, as previously described (Kuwana et al., 2025).

Fluorescence intensity profiles were obtained from raw fluorescence images using the line tool in CellSens software (Olympus). For each mature spore, a line was drawn along the longitudinal axis and the short axis, and the fluorescence intensity profile was recorded. The peak fluorescence intensity from each profile was used for quantitative analysis. Spores lacking GFP fusion constructs were used as negative controls to evaluate background fluorescence levels. Background fluorescence subtraction was not performed. The fluorescence intensity of spores lacking GFP fusion constructs was approximately 10–15 arbitrary intensity units under the imaging conditions used and was therefore considered negligible compared with the GFP signals detected in the fusion strains. Exposure times were optimized to avoid detectable autofluorescence in negative control samples and to prevent saturation of GFP signals. The same exposure settings were applied for comparisons between wild-type and *yabG*-deficient strains. Thirty spores were analyzed for each strain in each independent experiment, and three independent experiments were performed (*n* = 3). The longitudinal axis was defined as the long axis of the spore, whereas the short axis was defined as the axis perpendicular to the longitudinal axis at the center of the spore.

Fluorescence intensity measurements were performed using the original raw image data obtained from the microscope. For figure preparation, only uniform brightness adjustments were applied equally to all panels in Microsoft PowerPoint to improve visibility, and these adjusted images were not used for quantitative fluorescence analysis.

### 2.12 Statistical analyses

To compare the means of multiple groups and identify significant differences, we used two-way ANOVA followed by Tukey's multiple comparison test. Results with *P* ≤ 0.05 were considered statistically significant. All statistical analyses were performed using Microsoft Excel, as described previously (Kuwana et al., 2025).

## 3 Results

### 3.1 Identification of YabG protease-specific substrates using a combination of LC–MS/MS analysis and immunoblotting

To identify potential YabG-specific substrates, we previously performed a comparative proteomic analysis of wild-type and *yab*G-deficient spores using SDS–PAGE and LC–MS/MS (Kuwana et al., 2002; Kuwana, 2009). First, we prepared *B. subtilis* 168- and *yabG*-deficient spores. After separating the spore proteins via SDS–PAGE, the gels were divided into 64 blocks according to the band pattern of the wild-type strain and the resulting blocks were analyzed via LC–MS/MS (Kuwana et al., 2002). In this study, we reanalyzed and focused on protein bands specific to *yabG*-deficient spores to characterize novel candidate substrates in greater detail.

Twenty-three gel slices corresponding to protein bands specifically detected in *yabG*-deficient spores were excised from SDS–PAGE gels and analyzed by LC–MS/MS. These gel slices contained proteins with apparent molecular masses ranging from approximately 55 to 12 kDa, resulting in the identification of 83 proteins (Table 3 and Supplementary Figure 1). The number of amino acid residues, estimated molecular weights, and sigma factors involved in the regulation of gene expression were then determined using SubtiWiKi (http://subticom/uni-goettingen.de). Twenty-one proteins, including ArtP, CotB, CotC, CotE, CotF, CotG, CotR, CotS, CotU (YnzH), CotW, CotY, Mpr, PrsA, RsmH, SafA, SpoIVA, Vpr, YaaH, YdbO, YisY, and YxeE, were detected across multiple bands in *yabG*-deficient spores (Table 3, Supplementary Table 1). It should be noted that the detection of the same protein in multiple bands is a well-known artifact of in-gel digestion LC–MS/MS analysis, likely due to residual protein carryover between adjacent gel slices during sample handling or on the LC column, and should therefore be interpreted with caution. Next, the proteins detected in *yabG-*deficient spores were compared with those detected in the wild-type strain spore samples reported previously (Supplementary Table 1; Kuwana et al., 2002). We detected 29 proteins in *yabG*-deficient spores that were not detected in wild-type spores (gray columns in Supplementary Table 1). To identify candidate YabG substrates among these 29 proteins, we applied the following criteria: (1) expression under the control of SigE or SigK in mother cells and (2) known or predicted localization to the spore coat or basement layer. The remaining 25 proteins were excluded because they did not meet these criteria; for example, several were metabolic enzymes, ribosomal proteins, or proteins of unknown function unlikely to be spore coat components, and others were detected at molecular weights inconsistent with their predicted sizes, suggesting MS artifacts such as protein carryover between gel slices. Applying these criteria identified four candidate YabG-dependent proteins: GerPC (YisF), SpoVID, YeeK, and YsxE (Table 4). Among these, YeeK has already been identified as a YabG substrate by N-terminal sequencing analysis of protein bands specific to *yabG-*deficient spores (Takamatsu et al., 2000b) and subsequently characterized in detail (Takamatsu et al., 2009). We prioritized SpoVID for further characterization because previously established YabG substrates, including SpoIVA and SafA, are morphogenetic proteins involved in spore coat assembly (Takamatsu et al., 2000a), and SpoVID represents a major morphogenetic factor required for spore coat encasement. Although GerPC and YsxE were also identified as candidate YabG substrates, their detailed characterization remains an interesting subject for future investigation.

**Table 3:** Proteins identified in extracts from *B. subtilis yabG*-deficient spores.

**MM (kDa)**	Detected protein
55	SpoIVA
50	CotB, SafA (YrbA), SpoIVA, YaaH
45	HemAT (YhfV), SafA (YrbA), YaaH, YdhD, YhfE, YqgM
40	AprX, Bpr, CotH, CotI (YtaA), DacF, RacE, OxdD (YoaN)
38	CotS, DacB, MtlD, MurG, SafA (YrbA), YsxE
35	CotG, SpoIVB, SpoVID, Vpr, YhxC
34	CotB, CotG, RsmH (YlxA), YdbO
33	CotG, CotR (YvdO), CtaC, PrsA, YckD, YetM, YisY, YqhO
32	CotB, CotG, CotR (YvdO), FecC (YfmC), GDH, PrsA, RsmH (YlxA), YqfQ
31	CotG, RhiF (YesP), SodF, TasA (CotN), YjqC, YobI
29	PdaA (YfjS), SafA (YrbA), TcyA (YckK), YisY
28.5	ArtP (YqiX), Vpr, YfkD
28	ArtP (YqiX), CgeD, CotS, GerPC (YisF), RpsC, Tgl, YhbF
27	MelC (AmyC), Mpr, SsdC (YdcC), Vpr
26.5	CotE, CotF, LipC (YcsK), Mpr, YhfN, YrrT
24	CotE
22	CotE, CotF, CotY, QcrA, YdbO, YxeE
20	CotY, CotU (YnzH), YutJ, YxeE
19.5	CotF, CotY, CotZ, CwlJ, GerD, FtsH, RpsG, CotU (YnzH)
18	YeeK
17	CotC, SspE, YxeE
16	CgeA
14.5	CotB, CotC, CotW, DnaE, Mpr, RpmA, YabP, YfhD, YuzC
12	CotB, CotW, CwlD, RpmI, SspH (YfjU), Tlp, YxeE

MM indicates molecular mass of each band on an SDS–PAGE gel (Kuwana, 2009, YAKUGAKU ZASSHI 129: 1221–1225).

**Table 4:** YabG substrates and candidate substrates proteins.

Protein name	Localization	Regulator	Substrate status	Supporting evidence
SpoIVA	Basement layer	SigE	Confirmed substrate	Takamatsu et al. (2000a)
SpoVID	Basement layer	SigE	Putative substrate	This study
SafA	Basement layer	SigE	Confirmed substrate	Takamatsu et al. (2000a)
CotF	Inner coat	SigK	Confirmed substrate	Kuwana et al. (2006)
CotT	Inner coat	SigK	Confirmed substrate	Kuwana et al. (2006)
GerQ	Inner coat	SigK	Confirmed substrate	Kuwana et al. (2006)
YxeE	Inner coat	SigK	Confirmed substrate	Kuwana et al. (2007)
YabG	Inner coat	SigK	Self-cleaved	Yamazawa et al. (2022)
YeeK	Inner coat	SigK	Confirmed substrate	Takamatsu et al. (2009)
YsxE	Inner coat	SigE	Putative substrate	This study, McKenney and Eichenberger 2012)
GerPC	not determined	SigK	Putative substrate	This study

This table summarizes the confirmed and putative substrates of the YabG protease identified in this and previous studies. For each protein, its subcellular localization, known transcriptional regulation by SigE or SigK, substrate status, and supporting reports are indicated.

### 3.2 SpoVID is a putative new substrate for YabG protease

**Figure 1:**
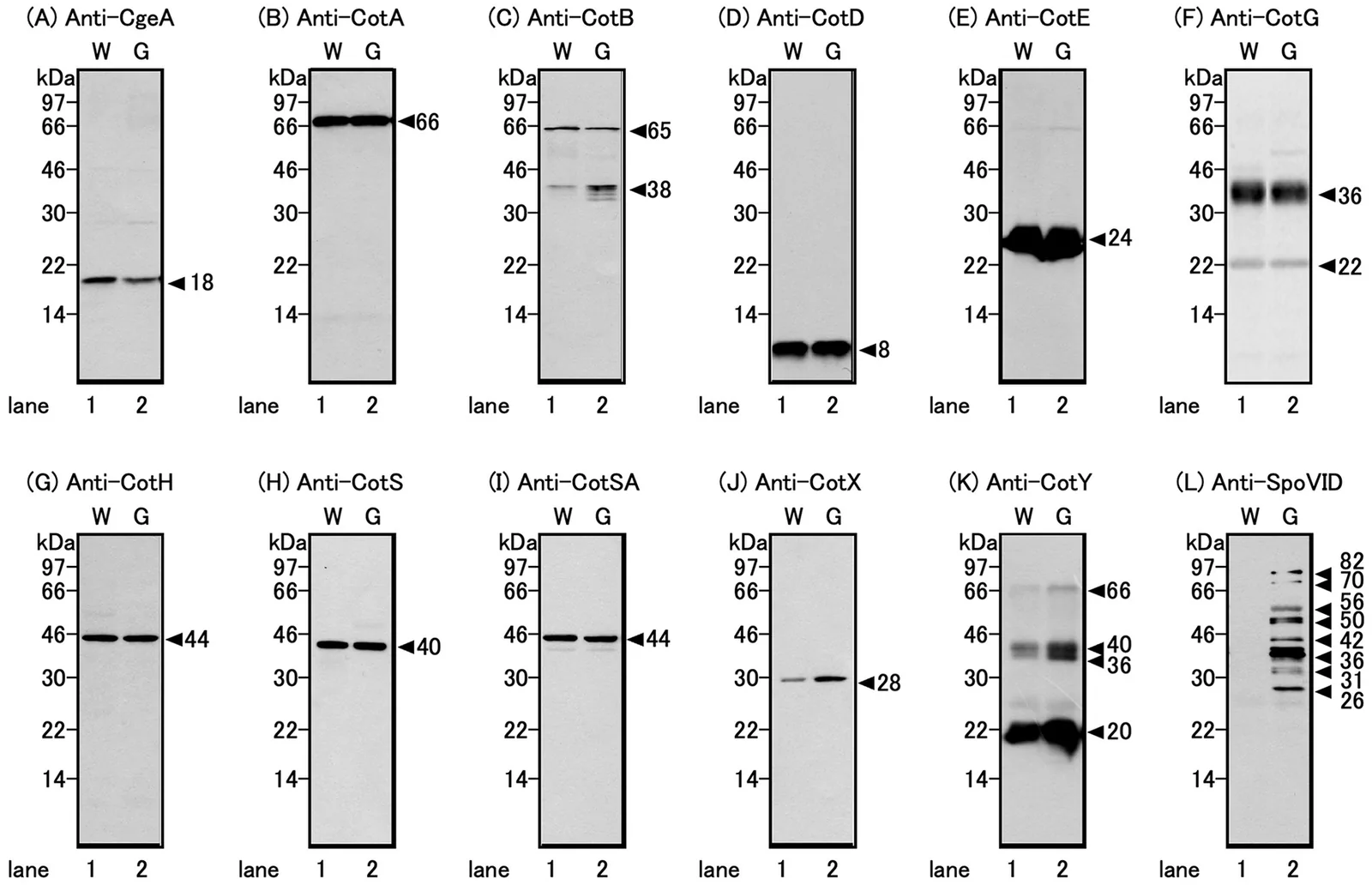
Comparison of spore coat proteins between the wild-type and *yabG-*deficient spores using immunoblotting analysis. Purified spore samples were prepared and analyzed using SDS–PAGE and immunoblotting with antibodies against CgeA **(A)**, CotA **(B)**, CotB **(C)**, CotD **(D)**, CotE **(E)**, CotG **(F)**, CotH **(G)**, CotS **(H)**, CotSA **(I)**, CotX **(J)**, CotY **(K)**, and SpoVID **(L)**. Lane 1: wild-type spores, lane 2: *yabG-*deficient spores. Arrowheads indicate the detected bands and molecular weights.

Since the substrates of YabG are coat proteins, immunoblotting analysis was performed on the major coat proteins to examine the substrate specificity of the YabG protease. Our previous studies analyzed the SpoIVA, SafA, CotF, CotT, GerQ, and YaaH proteins and reported SpoIVA, SafA, CotF, CotT, and GerQ to be substrates of the YabG protease (Takamatsu et al., 2000a; Kuwana et al., 2006). In this study, we examined spore coat proteins, such as CgeA, CotA, CotB, CotD, CotE, CotG, CotH, CotS, CotSA, CotX, CotY, and SpoVID in wild-type and *yabG*-deficient spores via immunoblotting with the corresponding antibodies (Figure 1). The detected band patterns were similar between the wild-type and *yabG*-deficient spores for 11 spore coat proteins: CgeA, CotA, CotB, CotD, CotE, CotG, CotH, CotS, CotSA, CotX, and CotY (Figure 1). Among these, proteins such as CotA, CotE, and CotH, which showed identical band patterns and intensities between the two strains, served as internal loading controls, confirming that equal amounts of protein were loaded per lane. The CotB proteins were detected as two bands with molecular weights of 65 and 38 kDa (Figure 1B). The intensity of CotB 38-kDa band, CotX 28-kDa band, and CotY 66, 40, and 36-kDa bands increased in *yabG*-deficient spores than in wild-type spores (Figures 1B, C, J, K). However, because no clear accumulation of processed CotB forms was observed, CotB itself was not considered a direct substrate of YabG. The estimated molecular weight of SpoVID was 64.8 kDa. No SpoVID band was detected in wild-type spore samples. In contrast, multiple SpoVID-derived bands ranging from approximately 82 to 26-kDa were detected in *yabG*-deficient spores (Figure 1L). The overall band patterns were reproducible across at least three independent experiments. Minor variations in the apparent molecular weights and intensities of some bands were observed between experiments, which were partly attributable to slight differences in SDS–PAGE migration and exposure time during chemiluminescence detection. These results suggest that SpoVID undergoes YabG-dependent proteolytic processing, possibly involving additional proteolytic events during spore maturation.

Next, *B. subtilis* strain 168 and *spoVID-*deficient cells were cultured in DS medium, and all cells were collected 5 h after initiation of spore formation. Protein samples of these cells were then analyzed using SDS–PAGE and detected using immunoblotting (Supplementary Figure 2). Analysis of the protein composition using SDS–PAGE and CBB staining showed similar band patterns between wild-type and *spoVID*-deficient cells (Supplementary Figure 2A). However, despite loading the same amount of sample, there were marked differences in the intensities of some bands. Specifically, bands at approximately 64, 48, 45, 40, 38, 34, 26, 23, and 15-kDa were less intense in *spoVID*-deficient cells than in wild-type cells. This result suggests that the absence of SpoVID affects the accumulation or stability of multiple sporulation-associated proteins, consistent with its role in coat protein assembly. Immunoblotting with the anti-SpoVID antibody detected a band at approximately 70 kDa in wild-type cells, which is slightly higher than the predicted molecular weight of 64.8 kDa, possibly reflecting anomalous migration on SDS–PAGE. No corresponding band was detected in *spoVID*-deficient cells (Supplementary Figure 2B), confirming antibody specificity. These results indicate that full-length SpoVID remains detectable in wild-type cells 5 h after sporulation initiation.

### 3.3 Proteolytic processing of SpoVID during spore maturation

**Figure 2:**
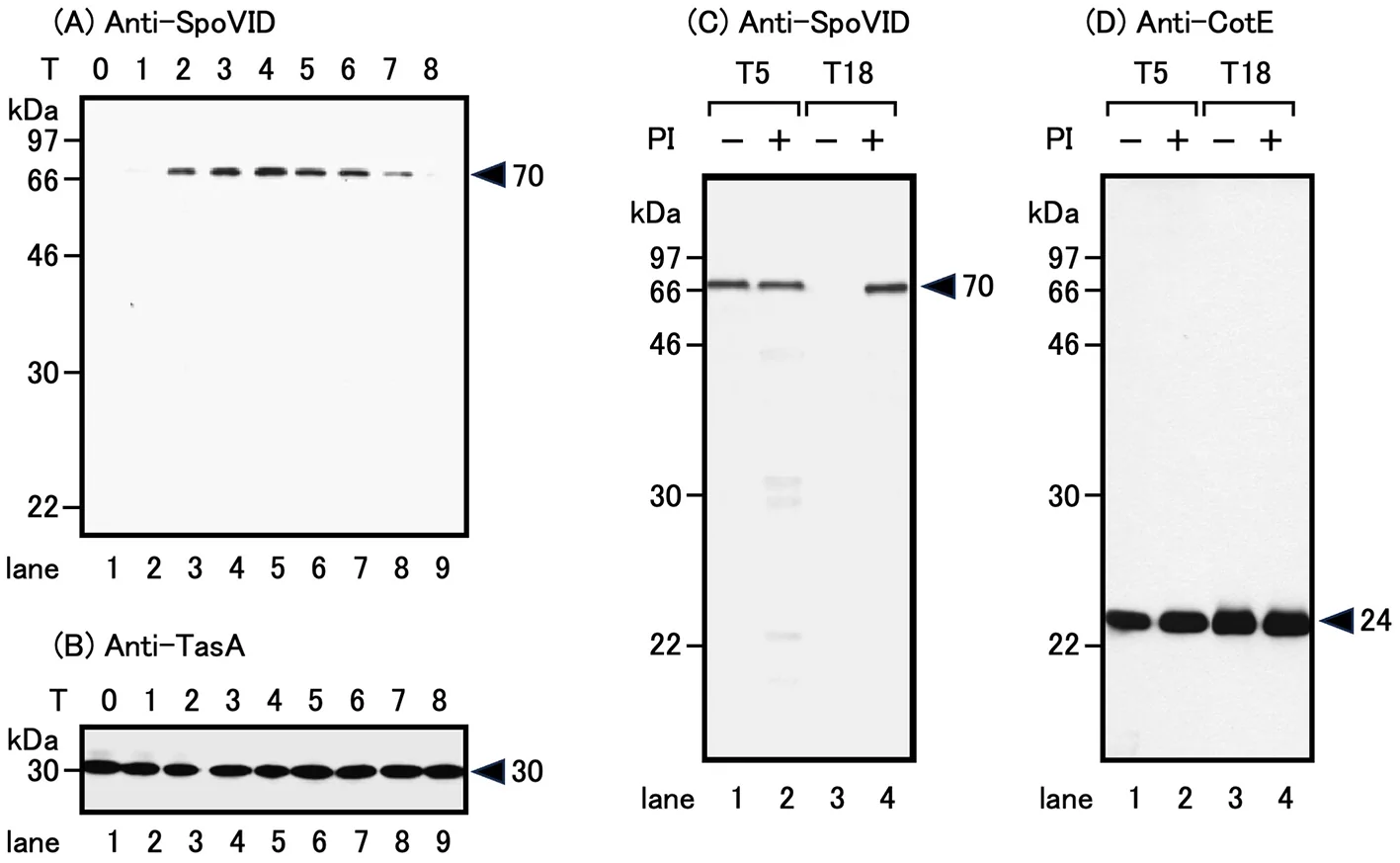
Temporal disappearance of SpoVID during sporulation and its inhibition by protease inhibitor treatment. *Bacillus subtilis* strain 168 cells were cultured in DS medium, and samples were collected hourly from T0 to T8 after the initiation of sporulation. Cell extracts were analyzed by immunoblotting using anti-SpoVID antibody **(A)** and anti-TasA antibody as a control **(B)** Lanes 1–9 correspond to samples collected at T0–T8, respectively. For protease inhibitor treatment, the cells were harvested at 5 and 18 h after the initiation of sporulation. A protease inhibitor cocktail (PI) was added to the culture 5 h after the start of sporulation. Samples with (+) or without PI (–) were analyzed via SDS–PAGE. SpoVID **(C)** and CotE **(D)** proteins were detected via immunoblotting with anti-SpoVID and anti-CotE antibodies, respectively. Lane 1: wild-type cells 5 h after the start of sporulation treated without PI, lane 2: wild-type cells 5 h after the start of sporulation treated with PI, lane 3: wild-type cells 18 h after the start of sporulation treated without PI, lane 4: wild-type cells 18 h after the start of sporulation treated with PI. Arrowheads indicate the detected bands and their molecular weights.

To determine when the full-length SpoVID protein became less detectable during sporulation, wild-type cells were cultured in DS medium and samples were collected hourly from T0 to T8 after sporulation initiation. Immunoblot analysis using anti-SpoVID antibody detected an approximately 70-kDa SpoVID band from T2 to T7 (Figure 2A). The intensity of the 70-kDa SpoVID band was markedly reduced at T7 and was barely detectable at T8, suggesting that SpoVID undergoes processing during the late stage of sporulation. As a control, immunoblotting with anti-TasA antibody detected an approximately 30-kDa band throughout sporulation (Figure 2B), consistent with the predicted molecular mass of TasA (28-kDa).

To test whether the loss of the 70-kDa SpoVID band was caused by proteolytic processing, sporulating cells were treated with a protease inhibitor (PI) at T5. This time point was selected because *yabG* transcription begins around T4–T5 under SigK control (Takamatsu et al., 2000b), allowing examination of SpoVID processing while SpoVID remained readily detectable and avoiding interference with pro-SigK processing, which is required for SigK-dependent gene expression, including *yabG*.

Samples were collected immediately before and after PI addition at T5 and after 13 h of further incubation (T18) from cultures grown in the absence or presence of PI. Proteins extracted from each cell were separated via SDS–PAGE, and the SpoVID protein was detected via immunoblotting using an anti-SpoVID antibody (Figure 2C). CotE was detected using an anti-CotE antibody as a control (Figure 2D). Five hours after the initiation of spore formation, the 70-kDa SpoVID protein was detected with or without the addition of PI. After 18 h of incubation following the initiation of spore formation, no 70-kDa SpoVID protein was detected in wild-type cells treated without PI, while a 70-kDa SpoVID protein was detected in wild-type cells treated with PI (Figure 2C). This result indicates that inhibition of protease activity prevents the disappearance of 70-kDa SpoVID during sporulation. Notably, although multiple SpoVID-derived bands were detected in mature *yabG*-deficient spores (Figure 1L), corresponding bands were not detected in wild-type cells during sporulation. In wild-type cells, the 70-kDa SpoVID band was detected from T2 to T7, barely detectable at T8, and not detected in mature spores. These results suggest that, following proteolytic processing, SpoVID-derived products may become poorly extractable, possibly because of incorporation into insoluble coat structures and/or protein complexes formed during spore maturation. Moreover, the 24-kDa CotE protein was detected with the anti-CotE antibody with or without the addition of PI, at 5 h or 18 h after the initiation of spore formation (Figure 2D). Because the protease inhibitor cocktail used in this study inhibits YabG as well as other proteases (Takamatsu et al., 2000a), these observations are consistent with a role for YabG in SpoVID processing; however, they do not demonstrate that YabG directly processes SpoVID, and the contribution of additional proteases cannot be excluded.

### 3.4 The proper localization of substrate spore coat proteins requires YabG

**Figure 3:**
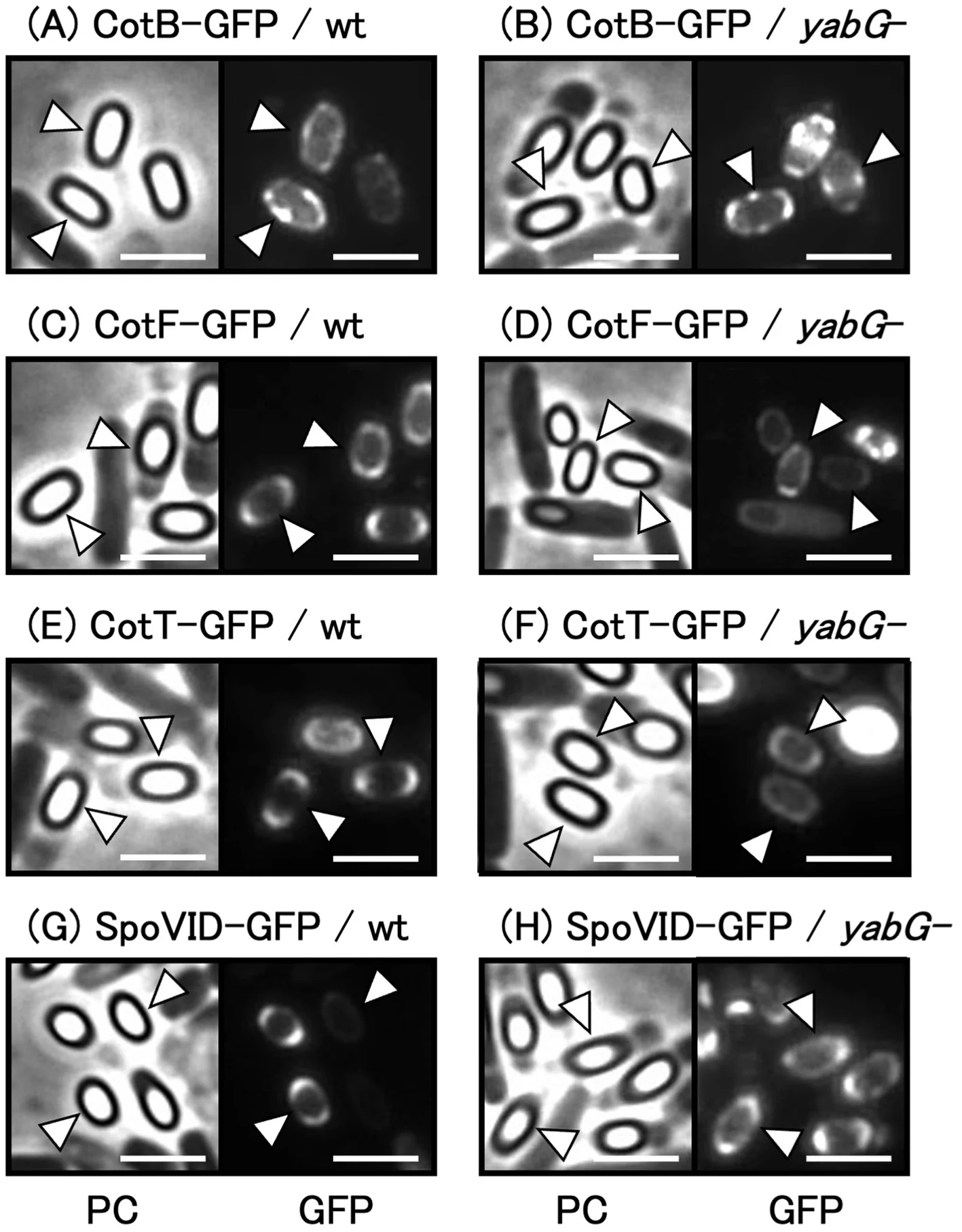
YabG affects the localization of CotF–GFP, CotT–GFP, and SpoVID–GFP. The cells were cultured in DS medium and analyzed using phase-contrast and fluorescence microscopy. The presence of CotB–GFP **(A, B)**, CotF–GFP **(C, D)**, CotT–GFP **(E, F)**, and SpoVID–GFP **(G, H)** was analyzed in wild-type **(A, C, E, G)** and *yabG*-deficient **(B, D, F, H)** strains. From left to right: Phase-contrast and GFP fluorescence images. Open arrowheads indicate mature spores. Scale bar: 2 μm.

We have previously reported that the YabG protease is involved in the spore coat localization of Tgl–GFP, YeeK–GFP, and YxeE–GFP in *B. subtilis* (Kuwana et al., 2006, 2007; Takamatsu et al., 2009). Among the proteins identified via LC–MS/MS analysis to be present in *yabG*-deficient spores were CotF, CotT, which are substrates of the YabG protease, the putative YabG substrate SpoVID, and CotB, which is not a substrate of the YabG protease (Figure 1). GFP fusion protein constructs were then generated for these proteins, and the localization of these spore coat proteins was analyzed in both wild-type and *yabG*-deficient strains (Figure 3). The wild-type and *yabG*-deficient strains harboring CotB–GFP, CotF–GFP, CotT–GFP, and SpoVID–GFP were then cultured in DS agar medium at 37 °C for 24 h. Prior to analyzing the localization of GFP fusion proteins, we evaluated whether available evidence supported the functionality of each construct. SpoVID–GFP has been reported to be functional in previous studies (Wang et al., 2009; de Francesco et al., 2012; Delerue et al., 2022). In this study, the SpoVID–GFP strain was able to form spores, suggesting that the GFP fusion did not severely interfere with the overall sporulation process. Imamura et al. (2010, 2011) reported that individual deletion of *cotB, cotF*, or *cotT* did not cause obvious defects in spore resistance, morphology, or germination. Therefore, it is difficult to evaluate whether GFP fusion affects the molecular function of CotB, CotF, or CotT based solely on these phenotypes. Phase contrast microscopy revealed that all GFP fusion strains sporulated and produced spores. To quantify the proportion of spores showing detectable GFP fluorescence, free spores in each fluorescence image were scored as GFP-positive or GFP-negative.

CotB–GFP fluorescence was detected in the coat of mature spores in both wild-type and *yabG-*deficient strains (Figures 3A, B). The proportion of GFP-positive spores was 57% (81/142) in wild-type and 76% (98/129) in *yabG*-deficient spores, suggesting that CotB is not a primary substrate of YabG, consistent with the immunoblotting results (Figure 1C). Occasionally, a strong transverse fluorescence signal at the mid-spore region was observed in *yabG*-deficient spore. CotF–GFP fluorescence was detected along the periphery of mature wild-type spores, with stronger fluorescence accumulation at both poles of the spores, forming cap-like structures (Figure 3C). In the *yabG*-deficient strain, the proportion of GFP-positive spores was markedly reduced than in wild-type spores (wild-type, 93%, 100/107; *yabG*-deficient, 41%, 54/133), and detectable fluorescence was distributed more uniformly along the forespore periphery without prominent polar accumulation (Figure 3D). In mature wild-type spores, CotT–GFP fluorescence was detected in a cap-like structure at both poles of the oval-shaped spores (Figure 3E). In *yabG*-deficient spores, the pronounced bipolar localization observed in wild-type spores was reduced. Although cap-like accumulation at both poles was still observed in some spores, CotT–GFP fluorescence was distributed more broadly around the spore periphery (Figure 3F). The proportion of GFP-positive spores was 88% (84/95) in wild-type and 80% (100/125) in *yabG*-deficient spores. SpoVID–GFP fluorescence was barely detectable in the majority of mature wild-type spores, with only 33% (49/149) of spores showing detectable fluorescence (Figure 3G). In the subset of wild-type spores showing detectable fluorescence, the signal was concentrated at both poles of the spore in a cap-like pattern. In contrast, SpoVID–GFP fluorescence was detected as cap-like structures at both poles in 80% (100/125) *yabG*-deficient spores, indicating that SpoVID–GFP accumulates at elevated levels and is retained at the poles in the absence of YabG (Figure 3H).

**Figure 4:**
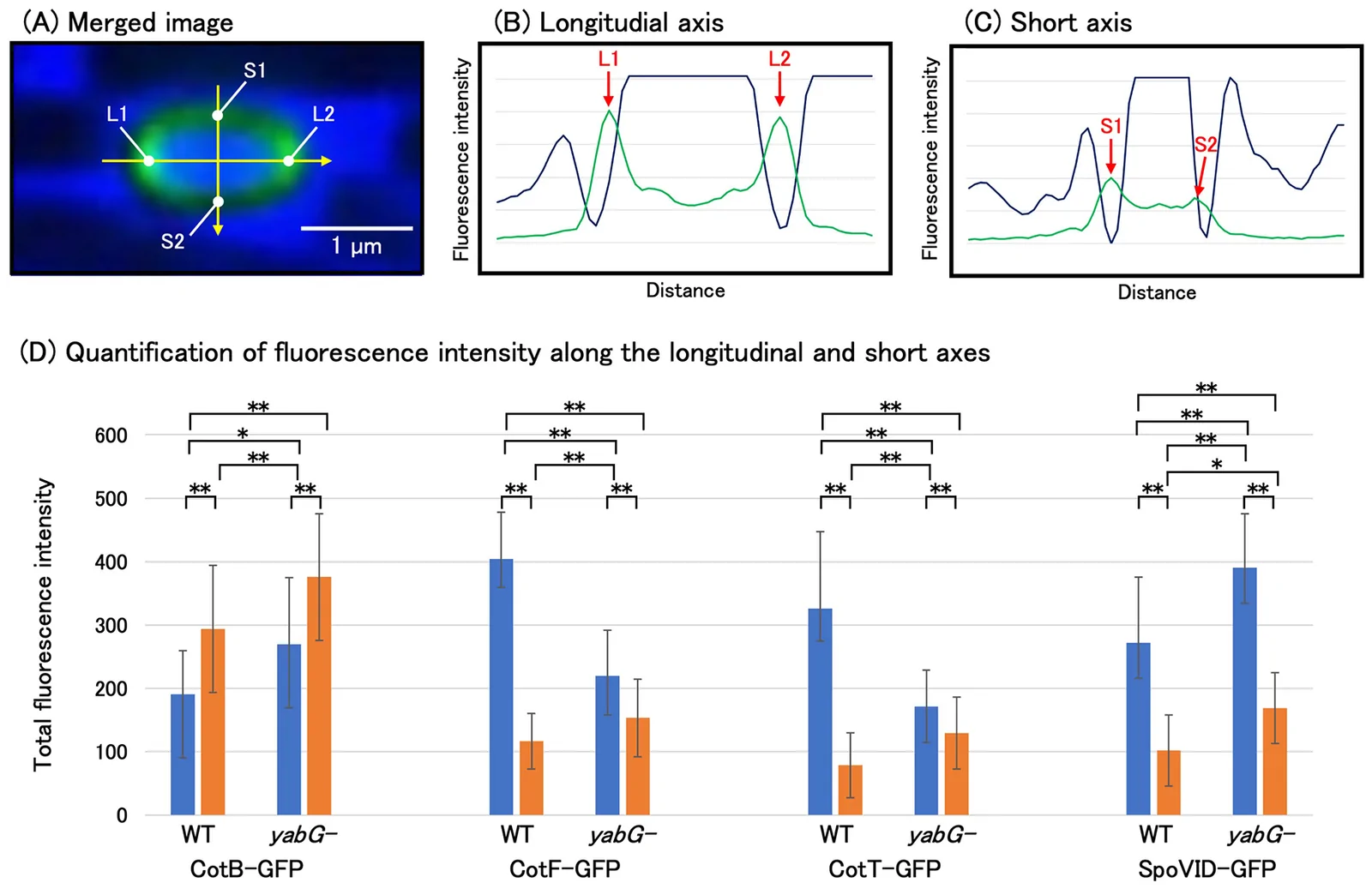
Localization and quantification of GFP fluorescence in spores. **(A)** Representative merged fluorescence microscopy images showing the method used for fluorescence intensity measurements. Blue indicates phase-contrast images, and green indicates GFP fluorescence. L1 and L2 indicate measurement points along the longitudinal axis, whereas S1 and S2 indicate measurement points along the short axis. Scale bar = 1 μm. **(B)** Representative fluorescence intensity profiles along the longitudinal axis of spores. The green and blue lines indicate GFP fluorescence and phase-contrast intensity, respectively. GFP fluorescence showed two positive peaks, designated L1 and L2. In the phase-contrast profile, negative peaks were observed around phase-bright spores due to the dark peripheral region surrounding the spores. **(C)** Representative fluorescence intensity profiles along the short axis of spores. The green and blue lines indicate GFP fluorescence and phase-contrast intensity, respectively. GFP fluorescence showed two positive peaks, designated S1 and S2. Negative peaks were observed in the phase-contrast profile around phase-bright spores. **(D)** Quantification of total fluorescence intensity along the longitudinal and short axes. Total fluorescence intensity was calculated as the sum of fluorescence intensities at L1 and L2 (longitudinal axis) or S1 and S2 (short axis) for CotB–GFP, CotF–GFP, CotT–GFP, and SpoVID–GFP in wild-type (WT) and *yabG*-deficient (*yabG*^−^) spores. Blue and orange bars indicate the summed fluorescence intensity along the longitudinal and short axes, respectively. Fluorescence intensity was quantified using cellSens software. Values are presented as mean ± SD. Asterisks indicate significant differences determined using two-way ANOVA followed by Tukey's multiple comparison test. *P < 0.05, **P < 0.01.

To further quantify the distribution pattern of fluorescence signals within individual spores, we measured the total fluorescence intensity along the longitudinal and short axes of mature spores for each GFP fusion protein in both wild-type and *yabG*-deficient backgrounds (Figure 4, *n* = 30). The fluorescence intensity for CotF–GFP, CotT–GFP, and SpoVID–GFP along the longitudinal axis was significantly higher than that along the short axis in both wild-type and *yabG*-deficient spores (*p* < 0.01; Figure 4D), indicating some degree of polar asymmetry regardless of YabG activity. However, the difference between fluorescence intensities for CotF–GFP and CotT–GFP along the longitudinal and short axes was significantly lower in *yabG*-deficient spores than in wild-type spores (Figure 4D), indicating reduced degree of polar asymmetry in the absence of YabG. The difference between longitudinal and short-axis fluorescence intensities for SpoVID–GFP was significantly greater in *yabG*-deficient spored than in wild-type spores (Figure 4D). In contrast, CotB–GFP exhibited significantly higher fluorescence intensity along the short axis than along the longitudinal axis in both genetic backgrounds (Figure 4D).

**Figure 5:**
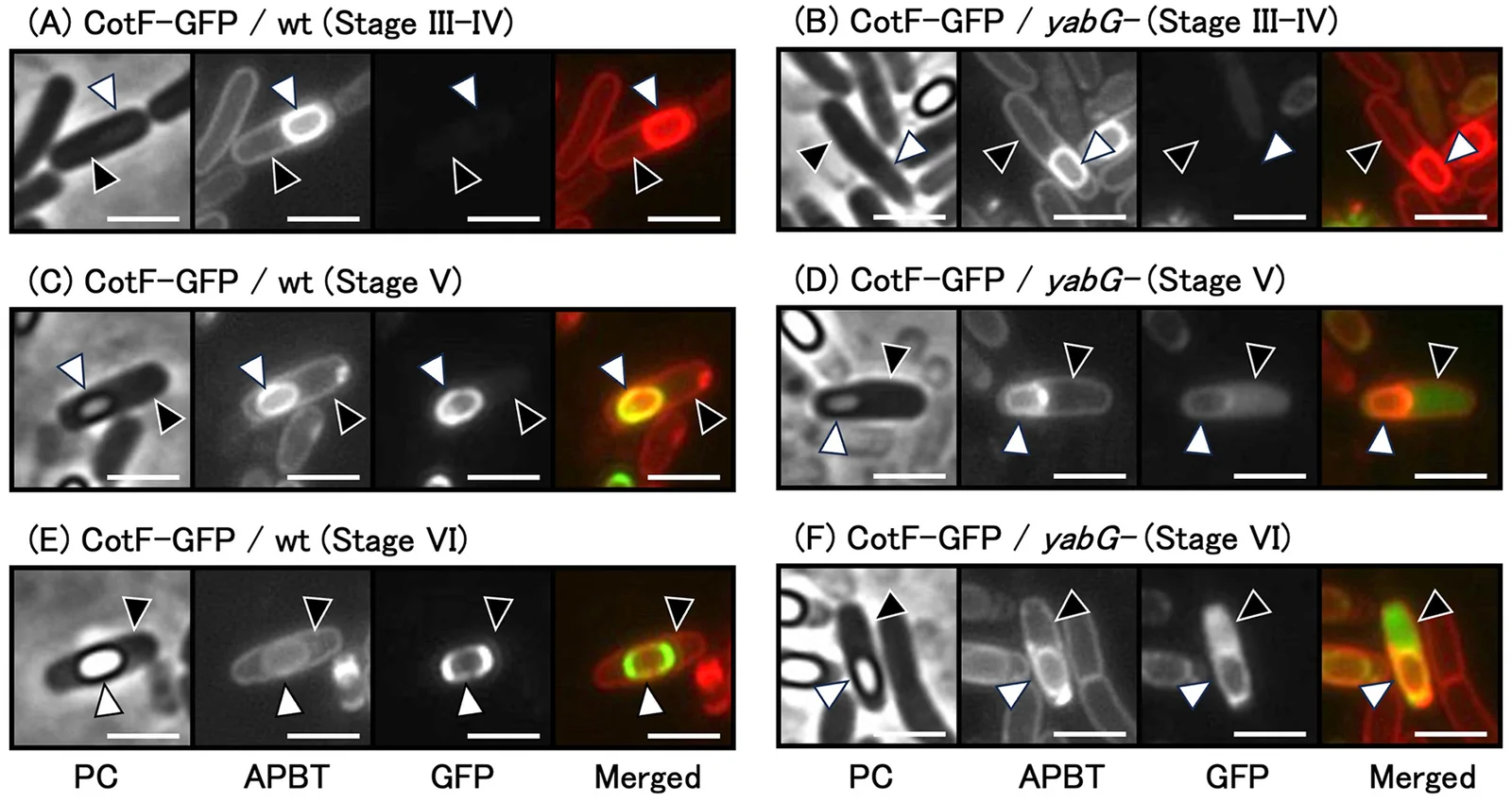
CotF–GFP fluorescence changes with the progression of sporulation in wild-type and *yabG-*deficient strains. Cells at sporulation stages III–IV **(A, B)**, V **(C, D)**, and VI **(E, F)** were analyzed. CotF–GFP cells with the wild-type background (A, C, and E) and *yabG*-deficient strains (B, D, and F) were suspended in a 2-(4-aminophenyl) benzothiazole (APBT) solution and observed using phase-contrast and fluorescence microscopy, respectively. From left to right: phase-contrast images, APBT fluorescence images, GFP fluorescence images, and merged images. Red represents APBT fluorescence and green represents GFP fluorescence. Open arrowheads indicate pre-spores or forespores. Closed arrowheads indicate mother cells. Scale bar: 2 μm.

**Figure 6:**
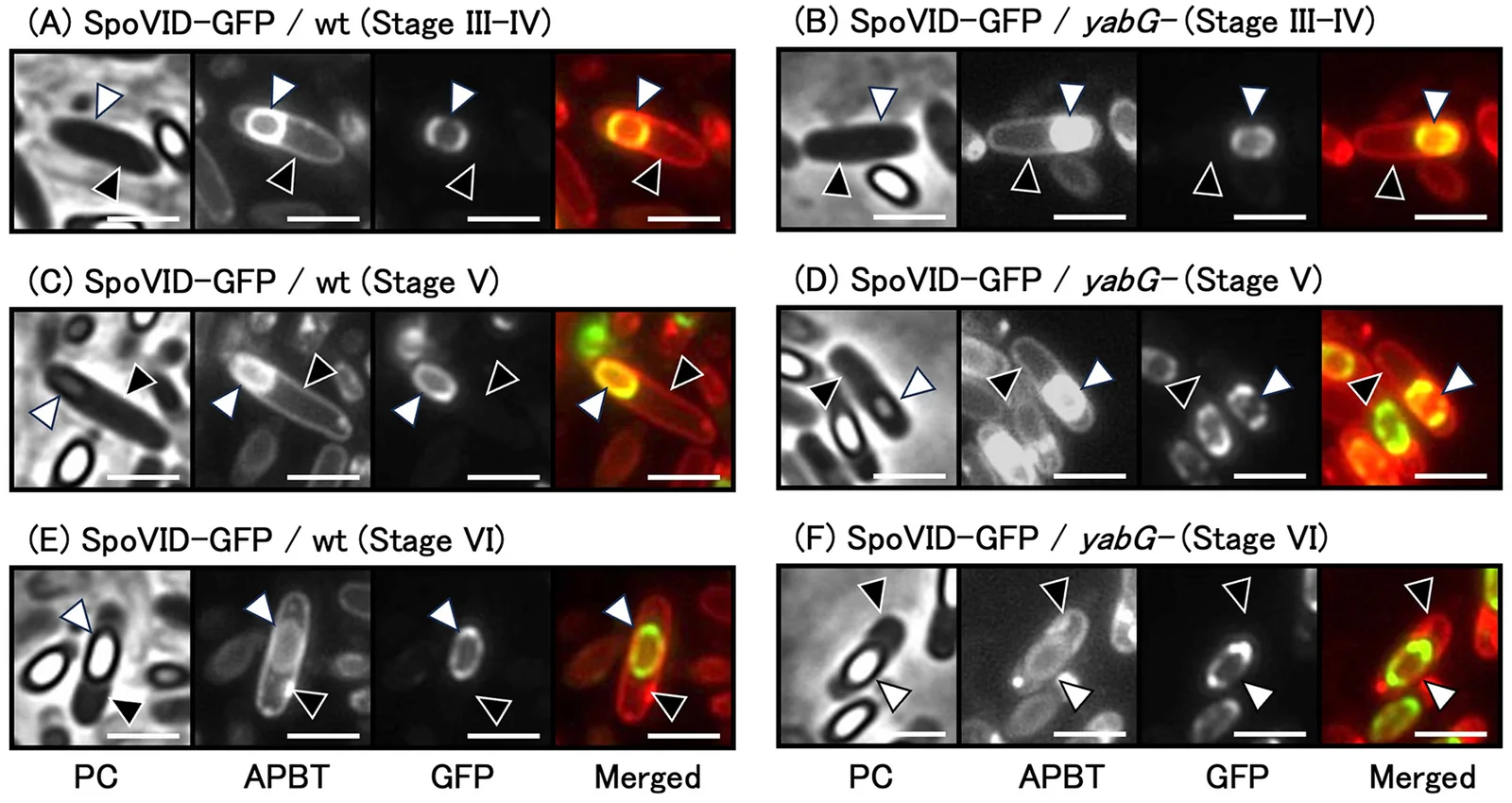
SpoVID–GFP fluorescence changes with the progression of sporulation in wild-type and *yabG-*deficient strains. Cells at sporulation stages III–IV **(A, B)**, V **(C, D)**, and VI **(E, F)** were analyzed. SpoVID–GFP cells with the wild-type background (A, C, and E) and *yabG*-deficient strains (B, D, and F) were suspended in a 2-(4-aminophenyl) benzothiazole (APBT) solution and observed using phase-contrast and fluorescence microscopy, respectively. From left to right: phase-contrast images, APBT fluorescence images, GFP fluorescence images, and merged images. Red represents APBT fluorescence and green represents GFP fluorescence. Open arrowheads indicate pre-spores or forespores. Closed arrowheads indicate mother cells. Scale bar: 2 μm.

Since CotF–GFP and SpoVID–GFP showed clear differences in fluorescence positivity between wild-type and *yabG*-deficient spores, we analyzed CotF–GFP and SpoVID–GFP fluorescence in cells at sporulation stages III–IV, V, and VI (Figures 5, 6). Sporulation stages were defined morphologically based on phase-contrast microscopy and APBT membrane staining: stage III, asymmetric division and forespore engulfment initiation; stage IV, forespore engulfment completion; stage V, cortex synthesis around the forespore; stage VI, spore coat assembly and forespore maturation; stage VII, mother cell lysis and mature spore release. These morphological criteria are consistent with those described previously (McKenney and Eichenberger, 2012; Kuwana et al., 2024). CotF–GFP fluorescence was not detected in stages III–IV in wild-type background or *yabG*-deficient cells (Figures 5A, B). In the wild-type background cells, CotF–GFP fluorescence was only detected along the periphery of the forespore at sporulation stage V and in a cap-like structure at the poles of the forespore at sporulation stage VI (Figures 5C, E). In the *yabG*-deficient strain, CotF–GFP fluorescence was detected around the forespore and in mother cells during stage V of sporulation (Figure 5D). CotF–GFP fluorescence was more strongly detected around the forespore and in the mother cells of *yabG*-deficient cells at stage VI of sporulation compared to that at stage V (Figure 5F). Furthermore, CotF–GFP fluorescence frequently accumulated at both poles of the forespore in wild-type cells at stage VI, whereas bipolar localization was less frequent in the *yabG*-deficient strain (Figures 4, 5E, F).

In wild-type background cells, SpoVID–GFP fluorescence was detected along the periphery of the forespore and in a cap-like structure at the poles of the forespore during sporulation stages III–IV (Figure 6A). However, SpoVID–GFP fluorescence in wild-type background cells was detected only along the periphery of the forespore during sporulation stages V and VI (Figures 6C, E). In *yabG*-deficient cells, SpoVID–GFP fluorescence was detected in cap-like structures at the poles of the forespores at sporulation stages III–VI (Figures 6B, D, F). These results indicate that the YabG substrates CotF, CotT, and SpoVID were synthesized and localized to the spore coat. Subsequently, they are processed or modified by YabG during spore maturation (Figures 2, 5, 6).

To further confirm that the changes in GFP fluorescence patterns reflect YabG-dependent proteolytic processing of the fusion proteins, we performed immunoblotting of mature spore extracts from CotF–GFP, CotT–GFP, and SpoVID–GFP strains with both the cognate antibody and an anti-GFP antibody (Supplementary Figure 3). For CotF–GFP, anti-CotF immunoblotting detected no band in wild-type spores but a band at approximately 48 kDa in *yabG*-deficient spores (Supplementary Figure 3A), consistent with the predicted molecular weight of the full-length CotF–GFP fusion protein (45.5 kDa). Anti-GFP immunoblotting detected bands at approximately 48, 40, and 28 kDa in wild-type spores, whereas a predominant 48-kDa band was detected in *yabG-*deficient spores (Supplementary Figure 3B). The 28-kDa band corresponds to free GFP. These results suggest that CotF–GFP undergoes proteolytic processing during spore maturation and full-length CotF–GFP accumulates in the absence of YabG.

For CotT–GFP, anti-CotT immunoblotting detected a faint band at approximately 39 kDa in wild-type spores and a markedly stronger band in *yabG*-deficient spores (Supplementary Figure 3C), consistent with the predicted molecular weight of the full-length CotT–GFP fusion protein (39.9 kDa). Anti-GFP immunoblotting detected bands corresponding to full-length CotT–GFP (~39 kDa) and free GFP (~28 kDa) in both wild-type and *yabG*-deficient spores, with an additional 16-kDa band detected in wild-type spores (Supplementary Figure 3D). These results suggest that CotT–GFP undergoes partial proteolytic processing during spore maturation and the full-length fusion protein accumulation increases in the absence of YabG.

For SpoVID–GFP, anti-SpoVID immunoblotting detected only a faint band at approximately 20 kDa in wild-type spores, whereas multiple stronger bands ranging from approximately 80 to 20 kDa were detected in *yabG*-deficient spores (Supplementary Figure 3E). Notably, anti-GFP immunoblotting detected a major band at approximately 28 kDa corresponding to free GFP and a faint band at approximately 18 kDa in wild-type spores (Supplementary Figure 3F), indicating that the SpoVID portion of the fusion protein is extensively processed during spore maturation, while the GFP portion remains relatively stable. In *yabG*-deficient spores, anti-GFP immunoblotting detected bands at approximately 65, 28, and 18 kDa, but no band corresponding to the predicted full-length SpoVID–GFP fusion protein (~91.8 kDa) was observed (Supplementary Figure 3F). These results suggest that SpoVID–GFP undergoes extensive but altered proteolytic processing in the absence of YabG, although the possibility that a fraction of the full-length fusion protein remained insoluble or was not efficiently extracted cannot be excluded.

### 3.5 YabG–GFP localizes to the basement layer and inner coat region of mature spores

**Figure 7:**
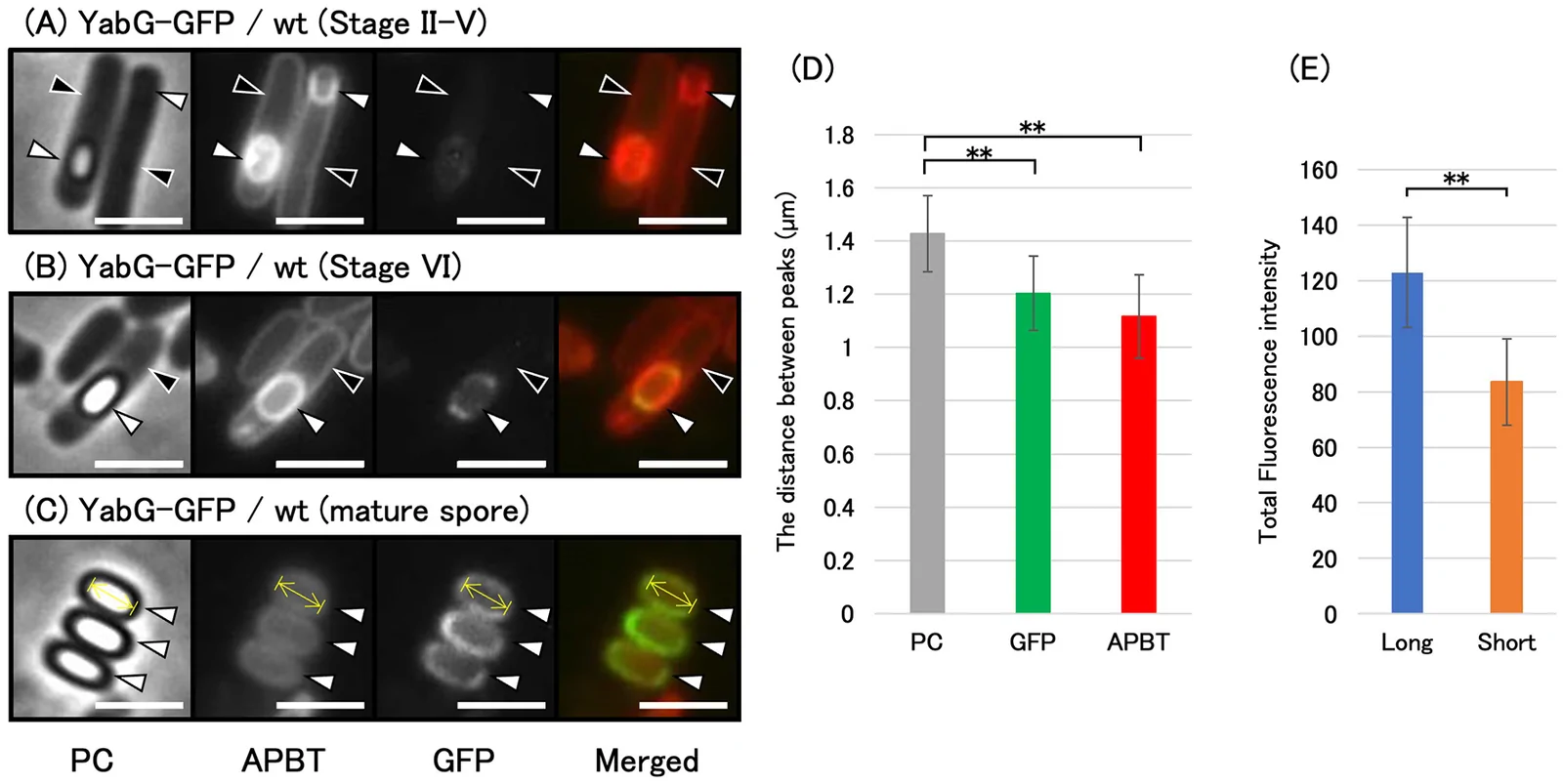
YabG–GFP co-localizes with the APBT signal in mature spores. YabG–GFP cells in sporulation stages II–V **(A)**, VI **(B)**, and VII **(C)** were suspended in a 2-(4-aminophenyl) benzothiazole (APBT) solution and observed using phase-contrast and fluorescence microscopy. From left to right: phase-contrast images, APBT fluorescence images, GFP fluorescence images, and merged images. Red represents APBT fluorescence and green represents GFP fluorescence. The yellow bar indicates the distance between the negative peaks in the phase-contrast images and the positive peaks in the fluorescence images. Open arrowheads indicate forespores. Closed arrowheads indicate mother cells. Scale bar: 2 μm. **(D)** The localization of YabG–GFP was determined via statistical analysis. Using the image analysis software CellSens, the distances between the negative peaks in the phase-contrast microscopy images and the positive peaks in the fluorescence microscopy images were measured for 30 spores each, as shown in **(C)**. Bar colors: red, APBT fluorescence; green, GFP signal; gray, phase-contrast measurements. The average values (μm) are shown in the graph. **(E)** Quantitative analysis of YabG–GFP fluorescence intensity along the longitudinal and short axes. Total fluorescence intensity was calculated as the sum of fluorescence intensities at L1 and L2 (longitudinal axis) or S1 and S2 (short axis) as defined in Figure 4. Fluorescence intensities were quantified from 30 mature spores shown in **(C)** using cellSens software. Blue and orange bars represent the summed fluorescence intensity along the longitudinal and short axes, respectively. Data are presented as the mean ± standard deviation. Asterisks indicate significant differences determined using two-way ANOVA, followed by Tukey's multiple comparison test to compare the conditions. **P < 0.01. Abbreviations: APBT, 2-(4-aminophenyl) benzothiazole; GFP, green fluorescent protein; SD, standard deviation; ANOVA, analysis of variance.

We conducted a detailed analysis of the subcellular localization of YabG–GFP in spore coats. Briefly, GFP fusion strains were grown on Schae?er's agar plates at 37 °C for 24 h, after which the cells were collected, stained with APBT, and observed under a phase-contrast and fluorescence microscope (Figure 7, *n* = 30). YabG-GFP fluorescence was barely detectable at sporulation stages II–V (Figure 7A). In contrast, during stage VI of sporulation in mature spores, YabG–GFP fluorescence was clearly visible at the forespore, and its signal largely overlapped with that of APBT (Figure 7C). We then measured the distance between the negative peaks of the mature spores in the phase-contrast images to define the length of the long axis of the mature spore. We also measured the distance between the positive APBT and YabG–GFP fluorescence peaks in the fluorescence images (Figures 7D). The average distance between the positive peaks of YabG–GFP fluorescence was similar to that of APBT, and no statistically significant difference was observed between the two measurements (Figure 7D). In contrast, the distance between APBT and YabG–GFP peaks were significantly smaller than the spore length determined by phase-contrast microscopy (*p* < 0.01). These results indicate that YabG–GFP localizes to a region corresponding to the APBT-stained layer in mature spores. The peak distance of YabG–GFP (≈ 1.2 μm) was not significantly different from that of APBT (≈ 1.1 μm), which stains the cortex and basement layer in mature spores (Kuwana et al., 2025). Since YabG does not contain a signal sequence or transmembrane sequence, localization to the outer spore membrane is unlikely. Furthermore, the peak distance of YabG–GFP (≈ 1.2 μm) was slightly smaller than that of YeeK–GFP (≈ 1.3 μm), which localizes to the inner spore coat (Kuwana et al., 2025), although the difference was not statistically significant. Taken together, these data suggest that YabG localizes to the basement layer and inner spore coat. This localization is further supported by the substrate profile of YabG: the confirmed YabG substrates SpoIVA and SpoVID are both basement layer proteins, while CotF, CotT, GerQ, YeeK, and YxeE localize to the inner spore coat (Imamura et al., 2010), and SafA, YsxE and YuzC also localize to the inner spore coat (McKenney et al., 2010; McKenney and Eichenberger, 2012; McKenney et al., 2013).

To further quantify the distribution pattern of fluorescence signals within individual spores, we measured the total fluorescence intensity along the longitudinal and short axes of mature spores for YabG–GFP fusion protein (Figure 7E, *n* = 30). The fluorescence intensity along the longitudinal axis was significantly higher than that along the short axis (*p* < 0.01), indicating that YabG accumulates at the both polar of the spore.

### 3.6 SpoVID affects YabG–GFP accumulation and localization during sporulation

SpoVID and SafA proteins are assembled on the surface of the forespore following the SpoIVA protein, and some spore coat proteins, such as CotE, are involved in the process of protein assembly on the forespore surface, as described previously (Driks, 1999; McKenney et al., 2013; Driks and Eichenberger, 2016). In sporulation stages IV–V, YabG–GFP fluorescence was detected around the forespore of *cotE*- and *gerE*-deficient strains, whereas it was detected in the mother cells of *spoIVA*-deficient strains (Takamatsu et al., 2000b).

**Figure 8:**
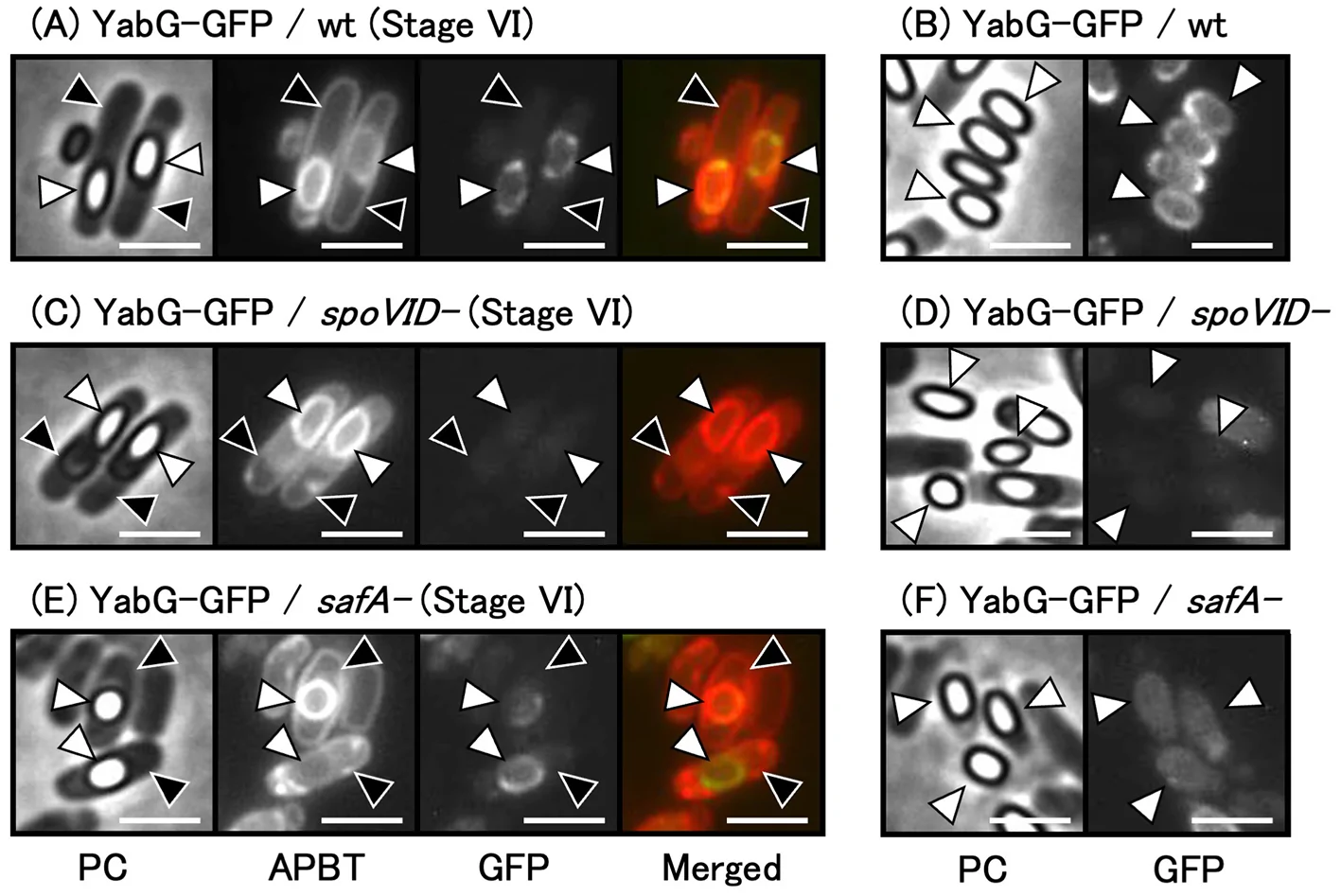
The localization of YabG–GFP depends on SpoVID and SafA. Cells at sporulation stage VI (A, C, and E) and mature spores (B, D, and F) were analyzed. YabG–GFP cells with wild-type **(A, B)**, *spoVID*-deficient **(C, D)**, and *safA*-deficient **(E, F)** strains were suspended in 2-(4-aminophenyl) benzothiazole (APBT) solution and observed using phase-contrast and fluorescence microscopy. From left to right: phase-contrast, APBT fluorescence, GFP fluorescence, and merged images. Red and green represent APBT and GFP fluorescence. Open arrowheads indicate the forespores (A, C, and E) or mature spores (B, D, and F). Closed arrowheads indicate mother cells. Scale bar: 2 μm.

In this study, we analyzed the localization of YabG–GFP at sporulation stage VI and in mature spores among wild-type, *spoVID-*, and *safA-*deficient cells during spore maturation (Figure 8). YabG–GFP fluorescence was detected around the forespore in wild-type and *safA*-deficient cells at stage VI of sporulation (Figures 8A, E), while it was barely detectable in *spoVID*-deficient cells (Figure 8C). YabG–GFP fluorescence was detected at the periphery of mature, wild-type spores (Figure 8B); however, it was weaker in mature, *safA-*deficient spores (Figure 8F). Furthermore, YabG–GFP fluorescence was barely detectable in *spoVID*-deficient spores (Figures 8C, D). Detectable YabG–GFP fluorescence was observed in 98% (100/102) wild-type spores, 13% (14/112) *spoVID*-deficient spores, and 79% (99/125) *safA*-deficient spores. These results indicate that YabG–GFP localization is more severely affected in the s*poVID*-deficient cells than in the *safA*-deficient cells, whereas a substantial fraction of YabG–GFP localization occurs independently of SafA. Notably, the reduction in YabG–GFP signal was more pronounced in *spoVID*-deficient cells than in *safA*-deficient cells, where YabG–GFP fluorescence was reduced but still detectable at the spore periphery (Figures 8E, F). These results suggest that the absence of SpoVID affects YabG–GFP accumulation and localization more severely than the absence of SafA. Owing to the essential role of SpoVID in spore coat encasement, the altered localization of YabG–GFP in *spoVID*-deficient cells is likely associated with the broader defects in coat assembly caused by the absence of SpoVID.

### 3.7 Structural analysis of *yabG*-deficient mature spores via transmission electron microscopy

**Figure 9:**
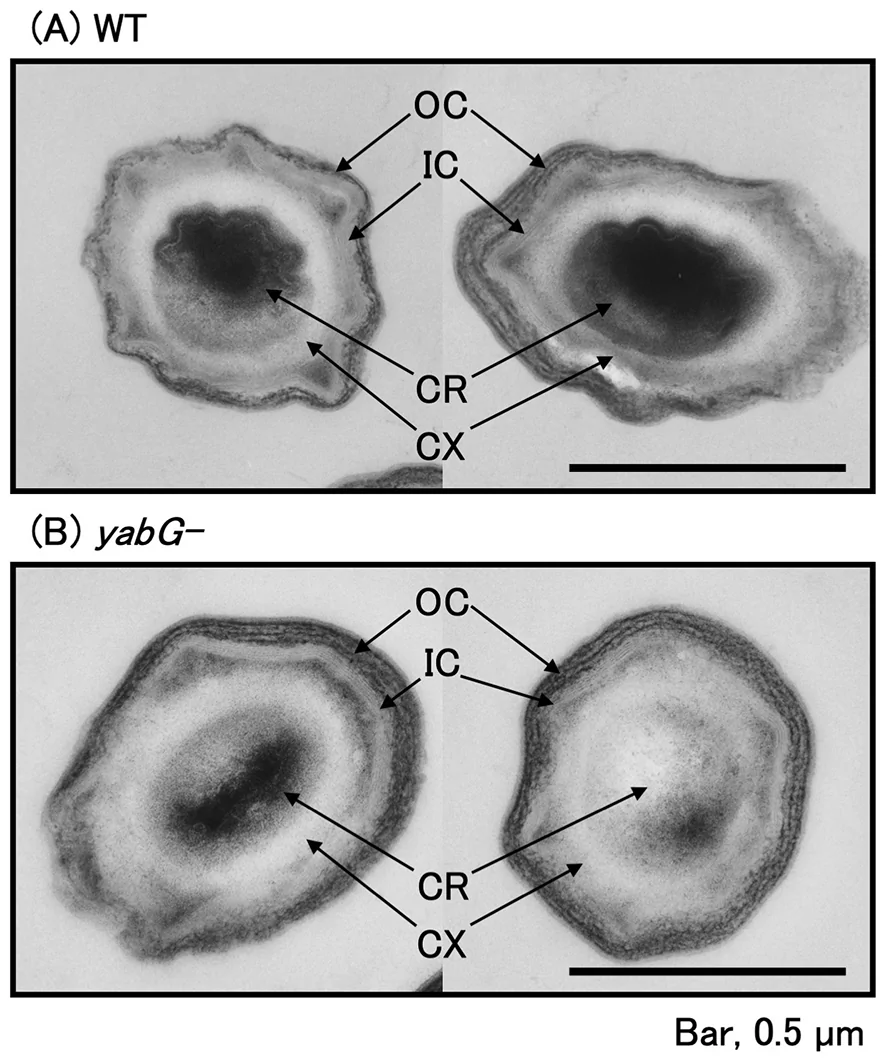
Transmission electron microscopy of *Bacillus subtilis* 168 and *yabG*-deficient spores. Cells were cultured in DS medium for 24 h and analyzed using transmission electron microscopy. Mature wild-type **(A)** and *yabG-*deficient **(B)** spores are shown. The outer spore coat (OC) with high electron density, inner spore coat (IC) with lamellar structures, cortex (CX), and core (CR) were observed in the wild-type and *yabG-*deficient spores. Scale bar: 0.5 μm.

Because we found that the YabG protease affects the fluorescence of SpoVID–GFP, we investigated the possibility that the YabG protease also affects the function of the SpoVID protein. Previously, *spoVID-*deficient cells have been reported to have incomplete spore coat formation and abnormal structures in the mother cells (Beall et al., 1993). Both wild-type and y*abG*-deficient spores were analyzed using transmission electron microscopy (Figure 9). TEM analysis was performed using purified spore samples. Among the ultrathin sections obtained, spore structures suitable for morphological evaluation were observed for six wild-type spores and six *yabG*-deficient spores (Figure 9 and Supplementary Figure 4). Although no obvious structural differences in spore coat morphology were consistently observed between the strains, the limited number of analyzable spores prevented reliable quantitative evaluation of morphological phenotypes. The outer spore coat (OC), which has high electron density, the inner spore coat (IC), with lamellar structures, the cortex (CX), and core (CR) were observed in wild-type spores (Figure 9A). Similar to the wild-type spores, *yabG*-deficient spores developed ICs and OCs with lamellar structures (Figure 9B). These observations suggest that no obvious defects in overall spore coat morphology were detected in *yabG-*deficient spores under the conditions examined.

## 4 Discussion

### 4.1 Identification of SpoVID as a putative YabG substrate

Proteomic and immunological analyses revealed that SpoVID is a putative substrate of the sporulation-specific protease YabG in *B. subtilis*. SpoVID was specifically detected in *yabG*-deficient spores, but not in wild-type spores, consistent with previous proteomic analyses (Kuwana et al., 2002; Kuwana, 2009). Immunoblotting further confirmed that SpoVID is synthesized as a 70-kDa protein during sporulation and becomes undetectable during spore maturation in wild-type spores, whereas multiple SpoVID-derived bands accumulated in *yabG*-deficient spores (Figure 1). The persistence of the full-length form of SpoVID in the presence of protease inhibitors suggests that proteolytic activity contributes to the loss of detectable full-length SpoVID during spore maturation. Together with the accumulation of SpoVID-derived fragments in *yabG*-deficient spores, these observations are consistent with a role for YabG in SpoVID processing. However, the absence of detectable SpoVID in wild-type spores may also reflect reduced extractability rather than proteolytic processing and one or more proteases in addition to YabG may generate the smaller SpoVID-derived fragments observed in the *yabG-*deficient spores.

### 4.2 Spatially regulated proteolytic activity by YabG during spore coat maturation

YabG is synthesized in the mother cell and undergoes autocleavage to generate the active form (Yamazawa et al., 2022). Since YabG activation in the mother-cell cytoplasm could potentially expose newly synthesized coat proteins to inappropriate proteolysis, spatial restriction of YabG within the developing coat may provide an effective mechanism to limit proteolytic activity to the appropriate stage and location of spore coat maturation.

YabG processes a subset of spore coat proteins that are primarily regulated by SigE and SigK (Takamatsu et al., 2000b; Takamatsu et al., 2009; Kuwana et al., 2006, 2007). Among these, SpoIVA and SafA are universally conserved, whereas SpoVID and GerQ are conserved among Bacillus species (Galperin et al., 2022). Our results suggest that the temporal expression and spatial localization of substrate proteins are key determinants of YabG specificity.

YabG localizes to the inner coat in a SpoIVA-, SpoVID-, and SafA-dependent manner (Figure 8; Takamatsu et al., 2000b), and its activity is likely restricted to this compartment. Furthermore, YabG was expressed under the control of SigK approximately 2 h after SigE activation, coinciding with late stages of coat assembly. Consistent with this model, quantitative fluorescence analysis demonstrated that YabG–GFP preferentially accumulated along the longitudinal axis of the forespore, similar to the localization patterns observed for SpoVID–GFP, CotF–GFP, and CotT–GFP. These observations support the idea that substrate accessibility within the same coat compartment contributes to efficient YabG-mediated proteolysis. Thus, YabG substrate selection is likely determined by not only cleavage-site recognition, but also spatial proximity between YabG and its substrates within the developing spore coat.

Although our results suggest that SpoVID undergoes YabG-dependent processing during spore maturation, the functional significance of this processing remains to be determined. Since SpoVID is required for proper spore coat assembly and encasement, YabG-mediated processing may contribute to the maturation of SpoVID-containing coat structures; however, this possibility remains to be tested. Consistent with this idea, altered localization patterns of CotF–GFP and CotT–GFP were observed in the *yabG*-deficient strain, suggesting that YabG activity contributes to the organization of specific coat proteins during spore coat maturation. However, whether SpoVID processing directly affects its interaction with other coat proteins or its molecular function remains unclear.

### 4.3 YabG-mediated remodeling of coat proteins

Fluorescence microscopy and immunoblot analysis of GFP fusion proteins demonstrated that YabG influences the spatial dynamics and processing of CotF and CotT during sporulation. In wild-type cells, these proteins localized to the forespore surface during coat formation but became less prominent in mature spores, whereas altered localization patterns and accumulation of full-length fusion proteins were observed in *yabG-*deficient cells (Figures 3, 4, and Supplementary Figure 3). These observations indicate that YabG-dependent proteolysis contributes to the rearrangement of coat proteins after their deposition instead of preventing their synthesis or initial localization. CotF–GFP was detected at the forespore surface during sporulation in both wild-type and *yabG*-deficient cells, indicating that initial recruitment occurs independently of YabG. However, the reduced frequency of GFP-positive spores, altered fluorescence distribution, and accumulation of full-length CotF–GFP in mature *yabG*-deficient spores suggest that YabG-dependent processing contributes to the proper localization and stable incorporation of CotF into the developing spore coat (Figures 3, 4, 5, and Supplementary Figure 3). A similar tendency was observed for CotT–GFP. Although CotT–GFP was recruited to the developing spore coat in both wild-type and *yabG*-deficient backgrounds, full-length CotT–GFP accumulation was higher in *yabG*-deficient spores (Supplementary Figure 3). Quantitative fluorescence analysis further showed that the degree of polar asymmetry was significantly reduced in the absence of YabG (Figure 4), suggesting that YabG-dependent processing contributes to the proper spatial organization of CotT during coat maturation. Previous studies have demonstrated that several YabG substrates, including SafA, GerQ, and YeeK, are also associated with Tgl-dependent cross-linking reactions (Kuwana et al., 2006; Fernandes et al., 2019). These observations suggest that YabG-dependent processing may represent one step in a broader coat maturation pathway involving additional modification enzymes.

In addition to coat-associated proteins such as CotF and CotT, YabG may also influence the maturation of morphogenetic scaffold proteins that organize coat assembly. SpoVID serves as a morphogenetic hub connecting SpoIVA in the basement layer with SafA/CotE-dependent coat structures and may coordinate coat assembly with cortex formation (Delerue et al., 2022). Once coat assembly is established, YabG-mediated SpoVID processing may contribute to the transition from coat assembly to coat maturation. Similar roles have been proposed for other YabG substrates, including SpoIVA and SafA (Takamatsu et al., 2000a). By processing these scaffold proteins after their assembly functions are fulfilled, YabG may facilitate structural rearrangement and stabilization of the developing spore coat. We propose that YabG functions as a late-stage remodeling protease that processes or remodels early morphogenetic scaffolds such as SpoIVA, SafA, and SpoVID once their primary assembly functions are completed.

Despite these molecular effects, *yabG*-deficient spores retained nearly normal morphology (Figure 9), resistance, and germination properties (Takamatsu et al., 2000b), suggesting that YabG is not essential for coat assembly itself but rather fine-tunes coat maturation. This distinguishes YabG from essential morphogenetic proteins such as SpoIVA, SafA, and SpoVID, which are indispensable for proper coat formation (Beall et al., 1993; Takamatsu et al., 1998).

### 4.4 Proposed model for YabG function in late sporulation

**Figure 10:**
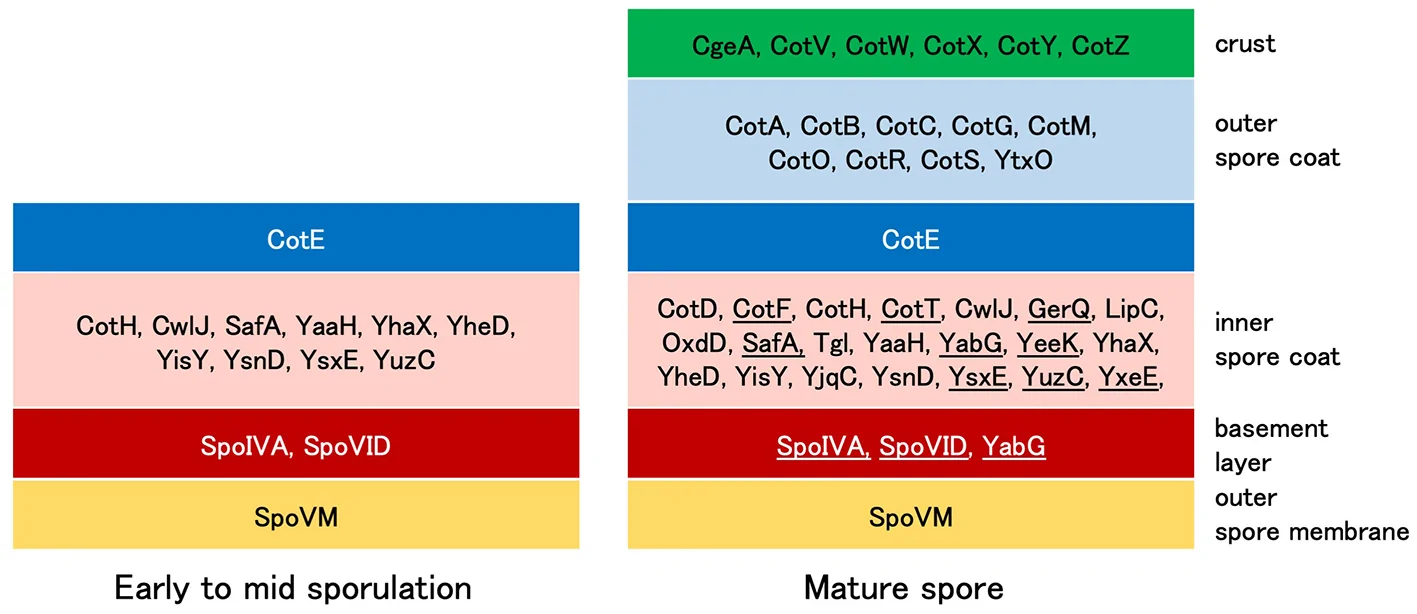
Model depicting the spatial and temporal regulation of spore coat protein assembly and YabG-dependent proteolysis during sporulation in *Bacillus subtilis*. This model illustrates the stepwise assembly and hierarchical organization of coat proteins in *B. subtilis* during sporulation. The protein composition and localization of the spore coat during early or mid-sporulation **(left)** and in mature spores **(right)** are shown. Proteins in the early or mid-sporulation stage are predominantly synthesized under the control of SigE, whereas proteins appearing later are mainly SigK-dependent. The morphogenetic proteins SpoIVA and SpoVID assemble to form the basement layer and are required for proper spore coat encasement. SafA and its processed forms are mainly associated with the inner spore coat. CotE, a SigE-dependent morphogenetic protein, localizes at the interface between the inner and outer coat layers and guides outer coat assembly. During late sporulation, YabG is produced and localized predominantly to the basement layer and inner spore coat, where it acts on proteins localized within these layers. YabG localization is strongly affected in *spoIVA*- and *spoVID-*deficient spores, whereas only partial reduction is observed in *safA*-deficient spores, suggesting that SpoIVA and SpoVID make a major contribution to YabG localization during coat encasement. YabG processes basement layer proteins (red), including SpoIVA and the putative YabG substrate SpoVID, as well as several inner coat proteins (pink), including SafA, CotF, CotT, GerQ, YabG, YeeK, and YxeE, after their localization to the spore surface. Proteins previously reported as YabG substrates or identified as putative YabG substrates in this study are underlined in the figure. In contrast, no YabG-dependent processing was detected for outer coat proteins (light blue; such as CotA, CotB, and CotG) or crust proteins (green; such as CotY and CgeA), based on the absence of molecular mass changes observed in immunoblot analyses from the present and previous studies. Similarly, no YabG-dependent processing was observed for SigE-dependent proteins such as CotE and YaaH. This spatial and temporal specificity suggests that YabG may contribute to the remodeling of the basement layer and inner coat during the final steps of coat maturation. The spatial localization of several coat proteins in this model is based on previous reports (Table 4), whereas the localization of YabG and YabG-dependent processing of the putative YabG substrate SpoVID, are supported by the present study.

Based on our results and previous findings, we propose a model for YabG-mediated coat remodeling (Figure 10). During early sporulation, SigE-dependent proteins such as SpoIVA and SpoVID assemble the basement layer, whereas SafA and associated proteins contribute primarily to inner coat formation. CotE organizes the boundary between the inner and outer coat layers. In later stages, SigK-dependent proteins, including YabG, are expressed. YabG localizes to the basement layer and inner coat region, where it acts on morphogenetic proteins and coat-associated proteins localized within these layers, including YabG substrates such as SpoIVA, SafA, CotF, CotT, GerQ, YeeK, and YxeE, as well as the putative substrate SpoVID and other YabG-associated proteins, during spore maturation. The apparent substrate preference of YabG is likely determined by both cleavage-site preference and spatial localization within the basement layer and inner coat. Consistent with this model, proteins localized predominantly to the outer coat or crust layers, such as CotE, CotB, CotY, and CgeA, were not identified as major YabG substrates in this study. Thus, YabG-mediated proteolysis represents a spatially confined maturation step that refines basement layer and inner coat architecture during late sporulation. These insights advance the understanding of how spatially and temporally coordinated proteolysis orchestrates spore coat maturation and differentiation in *B. subtilis*.

## Data Availability

The original contributions presented in the study are included in the article/Supplementary material, further inquiries can be directed to the corresponding author.

## Supplementary material

Supplementary Figure 1 SDS–PAGE and LC–MS/MS analysis of spore coat proteins in *yabG-*deficient spores. YabG spore-specific bands that differed from the SDS–PAGE bands of wild-type spores were cut out (gray), and the proteins were identified via LC–MS/MS analysis after in-gel digestion. The estimated molecular weights of the bands and the proteins identified from each band are shown. This figure is partially adapted from Kuwana (2009) with modifications. Reproduced in part with permission from Yakugaku Zasshi 129, 1221–1225. Copyright © 2009 The Pharmaceutical Society of Japan.

Supplementary Figure 2 SpoVID spore protein analysis by SDS-PAGE and immunoblotting. *Bacillus subtilis* strain 168 and *spoVID*-deficient strains were cultured in DS medium, and all cells were harvested 5 h after the initiation of spore formation. The wild-type (lane 1) and *spoVID-*deficient (lane 2) cells were analyzed via SDS–PAGE and stained with Coomassie Brilliant Blue (A) and SpoVID protein was detected by immunoblotting with anti-SpoVID antibodies (B). Lane 1: wild-type, lane 2: *spoVID*-deficient strain. Arrowheads indicate the detected bands and their molecular weights.

Supplementary Figure 3 Immunoblotting analysis of GFP fusion proteins in wild-type and *yabG*-deficient mature spores. Spore extracts were prepared from CotF–GFP (A, B), CotT–GFP (C, D), and SpoVID–GFP (E, F) strains in wild-type (lane 1) and *yabG*-deficient (lane 2) backgrounds. Immunoblotting was performed with anti-CotF (A), anti-CotT (C), anti-SpoVID (E), and anti-GFP (B, D, F) antibodies. Arrowheads indicate the detected bands and their molecular weights. The predicted molecular weights of the full-length fusion proteins are: CotF–GFP, 45.5 kDa; CotT–GFP, 39.9 kDa; SpoVID–GFP, 91.8 kDa. The 28-kDa band detected by anti-GFP corresponds to free GFP. Spores were harvested at T24.

Supplementary Figure 4 Additional transmission electron microscopy (TEM) of *Bacillus subtilis* 168 and *yabG*-deficient spores. Representative TEM images of wild-type (A) and *yabG*-deficient (B) spores. Four additional spores from each strain are presented to support the TEM observations shown in Figure 9. No obvious differences in overall spore morphology or coat layer organization were observed between wild-type and *yabG*-deficient spores under the tested conditions. Scale bar: 0.5 μm.

Supplementary Table 1 Proteins identified in extracts from *B. subtilis yabG-* spores.
