# Supplementary material for: Identification of SpoVID as a putative new substrate of the sporulation-specific protease YabG in Bacillus subtilis
Source: Front Microbiol. 2026 Aug 26;17:1838356. doi: 10.3389/fmicb.2026.1838356 (PMC13553755; doi:10.3389/fmicb.2026.1838356)
Supplement: Supplementary file 1 [file Table_1.pdf]

Supplementary Table 1. Proteins identified in extracts from *B. subtilis yabG-* spores.

Protein	BSU NO	Protein length (a.a.)	Apparent molecular mass (kDa)	Sigma factors	*yabG-* spore (kDa)	WT spore (kDa)
AprX	BSU_17260	442	47.74	SigA	40	42
ArtP (YqiX)	BSU_23980	255	28.16	-	28.5, 28	29
Bpr	BSU_15300	1433	154.34	-	40	ND
CgeA	BSU_19780	133	14.02	SigK	16	19
CgeD	BSU_19760	426	49.91	SigD, SigK	28	ND
CotB	BSU_36050	380	42.81	SigE	50, 34, 32, 14.5, 12	66 - 6.0
CotC	BSU_17700	66	14.64	SigK	17, 14.5	20 - 5.5
CotE	BSU_17030	181	20.83	SigE, SigK	26.5, 24, 22	59 - 5.5
CotF	BSU_40530	160	18.58	SigK	26.5, 22, 19.5	5.5, 5.0
CotG	BSU_36070	195	23.81	SigE, SigK	35, 34, 33, 32, 31	37 - 4.5
CotH	BSU_36060	362	42.65	SigE, SigK	40	42
CotI (YtaA)	BSU_30920	357	41.09	SigK	40	43, 42
CotR (YvdO)	BSU_34530	320	35.20	SigK	33, 32	33, 32, 31
CotS	BSU_30900	351	40.93	SigK	38, 28	42, 41, 16, 15
CotU (YnzH)	BSU_17670	86	11.40	SigK	20, 19.5	20, 19
CotW	BSU_11770	105	12.20	SigE, SigK	14.5, 12	15, 14
CotY	BSU_11750	162	17.74	SigE, SigK	22, 20, 19.5	52 - 15
CotZ	BSU_11740	148	16.00	SigE, SigK	19.5	18
CtaC	BSU_14890	356	39.94	-	33	32
CwlD	BSU_01530	237	26.86	SigE, SigG	12	ND
CwlJ	BSU_02600	142	16.22	SigE, SigK	19.5	19, 18
DacB	BSU_23190	382	42.92	SigE	38	39
DacF	BSU_23480	389	43.13	SigF, SigG	40	43
DnaE	BSU_29230	1115	125.14	-	14.5	29, 14
FecC (YfmC)	BSU_07520	315	34.87	-	32	32

FtsH	BSU_00690	637	70.76	SigA, SigF, SigM	19.5	19
GDH	BSU_03930	261	27.94	SigG	32	32
GerD	BSU_01550	185	20.97	SigF, SigG	19.5	20
GerPC (YisF)	BSU_10700	205	24.09	SigK	28	ND
HemAT (YhfV)	BSU_10380	432	48.60	SigD	45	ND
LipC (YcsK)	BSU_04110	213	23.46	SigK	26.5	26
MelC (AmyC)	BSU_30290	276	3022.00	SigA	27	ND
Mpr	BSU_02240	313	33.69	-	27, 26.5, 14.5	27, 26
MtlD	BSU_03990	373	40.31	SigA	38	ND
MurG	BSU_15220	363	39.82	SigA, SigE, SigM	38	ND
OxdD (YoaN)	BSU_18670	392	43.39	SigK	40	42, 41, 39
PdaA (YfjS)	BSU_07980	263	29.92	SigG	29	ND
PrsA	BSU_09950	292	32.36	-	33, 32	33, 32
QcrA	BSU_22560	167	18.59	SigA	22	19
RacE	BSU_28390	272	29.85	-	40	ND
RhiF (YesP)	BSU_06980	309	34.46	SigA	31	ND
RpmA	BSU_27940	94	10.23	-	14.5	13, 12
RpmI	BSU_28860	66	7.42	-	12	9.5
RpsC	BSU_01220	218	24.00	SigA	28	29
RpsG	BSU_01110	156	17.00	-	19.5	19
RsmH (YlxA)	BSU_15140	311	35.15	-	34, 32	ND
SafA (YrbA)	BSU_27840	387	43.07	SigE	50, 45, 38, 29	29
SodF	BSU_19330	281	33.32	SigE	31	26
SpoIVA	BSU_22800	491	55.01	SIgE	55, 50	20, 19
SpoIVB	BSU_24230	426	45.81	SigF, SigG	35	ND
SpoVID	BSU_28110	575	64.80	SigE	35	ND
SsdC (YdcC)	BSU_04630	338	42.03	SigE	27	28, 27, 26
SspE	BSU_08660	84	9.13	SigA, SigG	17	18, 17, 16
SspH (YfjU)	BSU_08110	59	6.73	SigA, SigG	12	ND
TasA (CotN)	BSU_24620	261	28.15	SigA	31	ND
TcyA (YckK)	BSU_03610	268	29.32	-	29	30
Tgl	BSU_31270	245	28.15	SigE, SigK	28	28

Tlp	BSU_18030	83	9.58	SigF, SigG	12	14, 10
Vpr	BSU_38090	806	85.42	SigH	35, 28.5, 27	ND
YaaH	BSU_00160	427	48.47	SigE, SigB	50, 45	52, 50, 48
YabP	BSU_00600	100	11.26	SigD, SigE, SigM, SigW, SigX	14.5	ND
YckD	BSU_03400	110	12.65	SigF, SigG	33	ND
YdbO	BSU_04540	290	31.33	-	34, 22	21, 8.0
YdhD	BSU_05710	420	48.80	SigE	45	44
YeeK	BSU_06850	145	15.78	SigK	18	ND
YetM	BSU_07230	369	40.82	-	33	ND
YfhD	BSU_08490	63	7.19	SigB, SigF, SigG	14.5	16, 14, 11
YfkD	BSU_07930	264	29.44	SigB, SigG	28.5	29
YhbF	BSU_08960	235	24.95	-	28	ND
YhfE	BSU_10200	346	38.58	-	45	43
YhfN	BSU_10290	426	48.71	-	26.5	ND
YhxC	BSU_10400	285	30.69	SigE	35	37
YisY	BSU_10900	268	30.41	SigE, SigK, SigG	33, 29	30
YjqC	BSU_12490	278	31.15	SigK	31	31
YobI	BSU_18970	1201	140.40	-	31	ND
YqfQ	BSU_25150	247	25.75	SigK	32	33
YqgM	BSU_24910	359	40.67	-	45	ND
YqhO	BSU_24510	291	32.71	-	33	ND
YrrT	BSU_27280	213	24.05	SigA	26.5	ND
YsxE	BSU_28100	341	40.41	SigE	38	ND
YutJ	BSU_32200	355	36.75	-	20	ND
YuzC	BSU_31730	122	14.05	SigE	14.5	6
YxeE	BSU_39580	121	14.57	SigK	22, 20, 17, 12	24 - 6.0

This table lists proteins detected by LC–MS/MS in purified spores of the *yabG*-deficient strain. The predicted molecular mass (kDa) was calculated based on the length of each protein. The predicted amino acid length, molecular mass, and regulatory information were obtained from SubtiWiki. "–" indicates that no annotation was available in SubtiWiki for the corresponding field. The molecular masses listed for *yabG*-deficient and wild-type (WT) spores represent apparent molecular masses estimated from the migration positions of protein bands on SDS–PAGE gels, rather than values directly measured by mass spectrometry. WT spore proteins are shown only for proteins also detected in *yabG*-deficient spores. WT data were obtained from Kuwana et al. (2002). Gray columns

indicate proteins detected specifically in *yabG*-deficient spores but not in WT spores. ND, not detected.
